# GDF11 slows excitatory neuronal senescence and brain ageing by repressing p21

**DOI:** 10.1038/s41467-023-43292-1

**Published:** 2023-11-17

**Authors:** Di-Xian Wang, Zhao-Jun Dong, Sui-Xin Deng, Ying-Ming Tian, Yu-Jie Xiao, Xinran Li, Xiao-Ru Ma, Liang Li, Pengxiao Li, Hui-Zhong Chang, Longqi Liu, Fan Wang, Yang Wu, Xiang Gao, Shuang-Shuang Zheng, Hui-Min Gu, Ya-Nan Zhang, Jian-Bin Wu, Fan Wu, Yonglin Peng, Xiao-Wen Zhang, Ren-Ya Zhan, Li-Xia Gao, Qiming Sun, Xing Guo, Xiao-Dong Zhao, Jian-Hong Luo, Ruhong Zhou, Lei Han, Yousheng Shu, Jing-Wei Zhao

**Affiliations:** 1 Department of Pathology of Sir Run Run Shaw Hospital, and Department of Human Anatomy, Histology and Embryology, System Medicine Research Center, NHC and CAMS Key Laboratory of Medical Neurobiology, Zhejiang University School of Medicine, 310058 Hangzhou, Zhejiang China; 2 Center of Cryo-Electron Microscopy, Zhejiang University, 310058 Hangzhou, Zhejiang China; 3 Department of Neurosurgery, Jinshan Hospital, Institute for Translational Brain Research, State Key Laboratory of Medical Neurobiology, MOE Frontiers Center for Brain Science, Fudan University, 201508 Shanghai, China; 4 BGI Research, 310030 Hangzhou, China; 5 The Global Scientific and Technological Innovation Center and the MOE Key Laboratory of Biosystems Homeostasis & Protection and Innovation Center for Cell Signaling Network, Life Sciences Institute, Zhejiang University, 310058 Hangzhou, Zhejiang China; 6 Key Laboratory of Systems Biomedicine (Ministry of Education), Shanghai; Center for Systems Biomedicine, Shanghai Jiao Tong University, 200240 Shanghai, China; 7 Zhejiang Provincial Key Laboratory for Cancer Molecular Cell Biology, Life Sciences Institute, Zhejiang University, Hangzhou, China; 8 Department of Neurosurgery, the First Affiliated Hospital, Zhejiang University School of Medicine, 79 Qingchun Road, 310003 Hangzhou, China; 9 Department of Neurology of the Second Affiliated Hospital, Interdisciplinary Institute of Neuroscience and Technology, Zhejiang University School of Medicine, 310020 Hangzhou, China; 10 Department of Biochemistry, and Department of Cardiology of Second Affiliated Hospital, Zhejiang University School of Medicine, Hangzhou, China; 11 Department of Neurobiology and Department of Anesthesiology, Sir Run Run Shaw Hospital, School of Medicine, Zhejiang University, 310058 Hangzhou, Zhejiang China; 12 NHC and CAMS Key Laboratory of Medical Neurobiology, MOE Frontier Science Center for Brain Research and Brain-Machine Integration, School of Brain Science and Brain Medicine, Zhejiang University, Zhejiang, China; 13 Institute of Quantitative Biology, College of Life Sciences, Zhejiang University, Hangzhou, China

**Keywords:** Neural ageing, Ageing

## Abstract

As a major neuron type in the brain, the excitatory neuron (EN) regulates the lifespan in C. elegans. How the EN acquires senescence, however, is unknown. Here, we show that growth differentiation factor 11 (GDF11) is predominantly expressed in the EN in the adult mouse, marmoset and human brain. In mice, selective knock-out of GDF11 in the post-mitotic EN shapes the brain ageing-related transcriptional profile, induces EN senescence and hyperexcitability, prunes their dendrites, impedes their synaptic input, impairs object recognition memory and shortens the lifespan, establishing a functional link between GDF11, brain ageing and cognition. In vitro GDF11 deletion causes cellular senescence in Neuro-2a cells. Mechanistically, GDF11 deletion induces neuronal senescence via Smad2-induced transcription of the pro-senescence factor p21. This work indicates that endogenous GDF11 acts as a brake on EN senescence and brain ageing.

## Abstract (web summary)

How excitatory neurons (EN) acquire senescence is unclear. Here, the authors show that GDF11 in ENs slows EN senescence, brain ageing, cognitive decline and maintains lifespan, revealing a mechanism underlying EN senescence and brain ageing.

## Introduction

Ageing drives cognitive decline and mental fragility during healthy ageing life^1^ and is also the most prominent single risk factor for neurodegenerative diseases^2^. Cellular senescence is a fundamental mechanism underlying organismal ageing and can occur not only in proliferative cells^3^ but also in post-mitotic neurons^4^ of the central nervous system (CNS). Indeed, post-mitotic neuronal senescence is presented in the aged brain of multiple species including mouse^5^ and human^6,7^. Importantly, neuronal senescence could be the main culprit of human organismal ageing^6,8^. Senescent post-mitotic neurons shift their activity to strengthen their secretory function and acquire the senescence-associated secretory phenotype (SASP)^8^. However, how senescent neurons negatively influence other cells through the SASP is just beginning to be explored experimentally^9^. Ageing drives distinct transcription programs in a cell-type-dependent manner in the brain^10^, and a recent study reported that the predominant senescent cell type in the postmortem aged human brain is the excitatory neuron (EN)^7^. With chronological ageing, the activity of the EN in the brain increased while inhibition of the EN activity extends lifespan in *C. elegans*^11^. These studies have highlighted the EN as the major cell type which contributes to ageing of not only the brain but also the whole body. However, these questions remain unknown. (1) How does the EN itself in the brain get senescent? (2) Whether induction of cellular senescence of the EN is sufficient to cause brain ageing, cognitive decline and even affects lifespan? It is imperative to understand how brain ages by answering these questions.

Here we show that growth differentiation factor 11 (GDF11), a putative systemic rejuvenation factor^12–14^, was predominantly expressed in the EN in the adult mouse, marmoset and human brain. In vivo selective deletion of GDF11 in the post-mitotic EN caused EN senescence and hyperexcitability, reduced their synaptic inputs, impaired object recognition memory and social cognition and shortened lifespan in mice. In vitro knocking-out GDF11 induced cellular senescence. Mechanistically, loss of GDF11 induces neuronal senescence via Smad2-induced upregulation of p21. This study uncovers a molecular mechanism that GDF11 slows excitatory neuronal senescence, brain ageing and maintains lifespan.

## Results

### GDF11 is expressed in the excitatory neurons of the adult brain

**Fig. 1:**
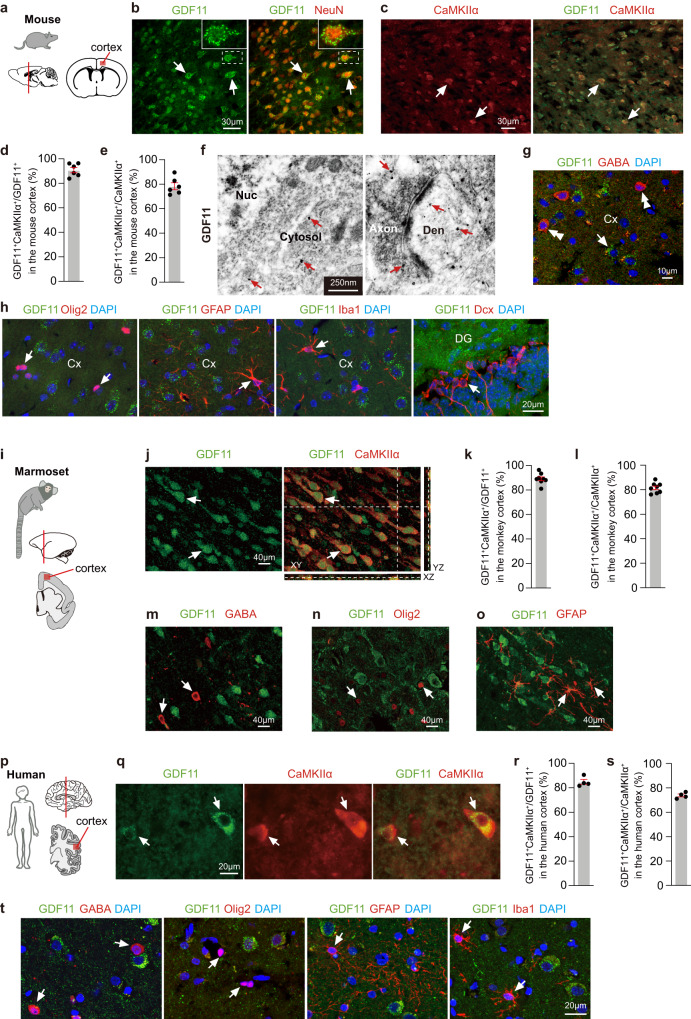
GDF11 is predominantly expressed in the EN in the adult mouse, marmoset and human brain. **a**–**e** Schematic diagrams of the brain of mouse (**a**), and the red box in the cerebral cortex shows the location where the images were taken. Immunofluorescence double labelling (**b**, **c**, 2 double-labelled neurons are indicated as examples in (**b**, **c**)) and quantification (**d**, **e**, *n* = 6 images from 3 mice) of GDF11 (green, **b**) and NeuN (red, **b**) or GDF11 (green, **c**) and CaMKIIα (red, **c**) in the cerebral cortices of the mice aged 3 months (3 M). **f** Representative images of immuno-electron microscopy (Immuno-EM) of GDF11 labelled with nanogold particles (there are many GDF11 labelled black dots and only some examples are indicated with red arrows) in the cerebral cortex of the mice aged 3 M (*n* = 3 mice). Nuc, nucleus; Den, dendrite. **g** Immunofluorescence double labelling of GDF11 (green, arrow) and GABA (red, double arrowheads) (*n* = 3 mice). **h** Immunofluorescence double labelling of GDF11 (green) together with Olig2 (red, left), GFAP (red, middle), Iba1 (red, middle) in the cerebral cortex (Cx) and Dcx (red, right) in the dentate gyrus (DG) of the mice aged 3 M (*n* = 3 mice). The GDF11 negative cells are indicated by arrows in (**h**). **i** Schematic diagrams of the brain of the marmoset (one aged 62 M and another aged 70 M), and the red box in the cerebral cortex shows the location of the images (*n* = 2 marmosets). **j**–**o** Immunofluorescence double labelling (**j**, **m**, **n**, **o**) and quantification (**k**, **l**) of GDF11 (green) together with CaMKIIα (red, **j**, **k**, **l**, 2 double-labelled neurons are indicated as examples in (**j**); *n* = 8 images from 2 marmosets) or GABA (red, **m**), Olig2 (red, **n**) or GFAP (red, **o**). The GDF11 negative cells are indicated by arrows in (**m**, **n**, **q**). **p** Schematic diagrams of the human brain. The red box in the cerebral cortex shows the location of the images. **q**–**s** Immunofluorescence double labelling (**q**, male patient aged 24 years (Y) and female patient aged 23Y diagnosed with intractable epilepsy and the focus of epileptic cortices had to be removed surgically) and quantification (**r**, **s**, *n* = 4 patients, male patient aged 23Y, male patient aged 52Y, female patient aged 54Y and male patient aged 60Y suffered brain injury) of GDF11 (green) together with CaMKIIα (red) in the cerebral cortex of patients and 2 double-labelled neurons are indicated by arrows in (**q**). **t** Immunofluorescence double labelling of GDF11 (green) together with GABA (red, left), Olig2 (red, middle), GFAP (red, middle) and Iba1 (red, right) in the cerebral cortex of patients (*n* = 4 patients). The GDF11 negative cells are indicated by arrows in (**t**). Scale bars, as shown on the images, 30 μm (**b**, **c**), 250 nm (**f**), 10 μm (**g**), 40 μm (**j**, **m**, **n**, **o**), 20 μm (**h**, **q**, **t**). Data are presented as mean ± SEM. Source data are provided with this paper.

The specificity of 4 anti-GDF11 antibodies was carefully characterized here (Supplementary Fig. 1a–d and Supplementary Table 1) and partially in previous studies^15,16^, and was further validated by using the cerebral cortices of the conditionally GDF11 knockout mice, GDF11^cKO^ mice (GDF11^f/f^; Ca^2+^/calmodulin-dependent protein kinase II alpha-Cre, abbreviated to CaMKIIα-Cre), in which GDF11 was deleted exclusively in the CaMKIIα^+^ EN (Supplementary Fig.1e). Using immunofluorescence of this specific anti-GDF11 antibody, mouse anti-GDF11 antibody (R&D, MAB19581), we show that in the adult brain of mouse aged 3 months (M) (Fig. 1a), GDF11 was widely expressed in neurons in the cerebral cortex (Fig. 1b) and other parts of the CNS, consistent with a previous study^17^. We further found that GDF11 was predominantly expressed in the CaMKIIα^+^ EN in the cerebral cortex (Fig. 1c) of the adult mouse: about 90% GDF11^+^ neurons were CaMKIIα^+^ (Fig. 1d) while about 80% CaMKIIα^+^ neurons were GDF11^+^ (Fig. 1e). Immuno-electron microscopy (immuno-EM) further revealed that GDF11 was localized to the cytoplasm, the Golgi complex, the excitatory presynaptic axonal terminals and their postsynaptic dendrites (Fig. 1f and Supplementary Fig. 2a, b). GDF11 was found neither in γ-aminobutyric acid (GABA) positive inhibitory neurons (Fig. 1g), Olig2^+^ oligodendrocyte lineage cells (Fig. 1h), glial fibrillary acidic protein (GFAP) positive astrocytes (Fig. 1h), Ionized calcium-binding adapter molecule 1 (Iba1) positive microglia (Fig. 1h), doublecortin (Dcx) positive neuronal progenitors (Fig. 1h), nor at the inhibitory synapses using immuno-EM (Supplementary Fig. 2c).

To explore whether the expression of GDF11 in the EN conserves across different species, we found that in the cerebral cortices of two marmosets aged 62 M and 70 M, respectively (Fig. 1i), GDF11 was expressed in the soma and main processes of CaMKIIα^+^ EN (Fig. 1j). Again, about 90% GDF11^+^ neurons were found to be CaMKIIα^+^ (Fig. 1k), and 80% CaMKIIα^+^ neurons expressed GDF11 (Fig. 1l), highly consistent with our results of the mouse (Fig. 1d, e). GDF11 was not seen in GABA^+^ inhibitory neurons (Fig. 1m), Olig2^+^ oligodendrocyte lineage cells (Fig. 1n), or GFAP^+^ astrocytes (Fig. 1o). Furthermore, using cortical tissues of 4 patients, aged from 23 to 60 years of both male and female, who suffered acute brain injury and 2 patients, aged 23 and 24 years respectively, who were diagnosed with intractable epilepsy and the focus of epileptic cortices had to be removed surgically, we found that in the peri-lesion and peri-epileptic cortices, GDF11 was predominantly expressed in the CaMKIIα^+^ EN in the cerebral cortex of the adult human brain (Fig. 1p–t), similar to that of GDF11 in the cerebral cortices of the mouse and the marmoset in both the pattern and the quantification. Collectively, our data indicate that GDF11 is predominantly expressed in the EN in the adult mouse, marmoset and human brain.

### Loss of GDF11 in excitatory neurons causes their own senescence and brain ageing

**Fig. 2:**
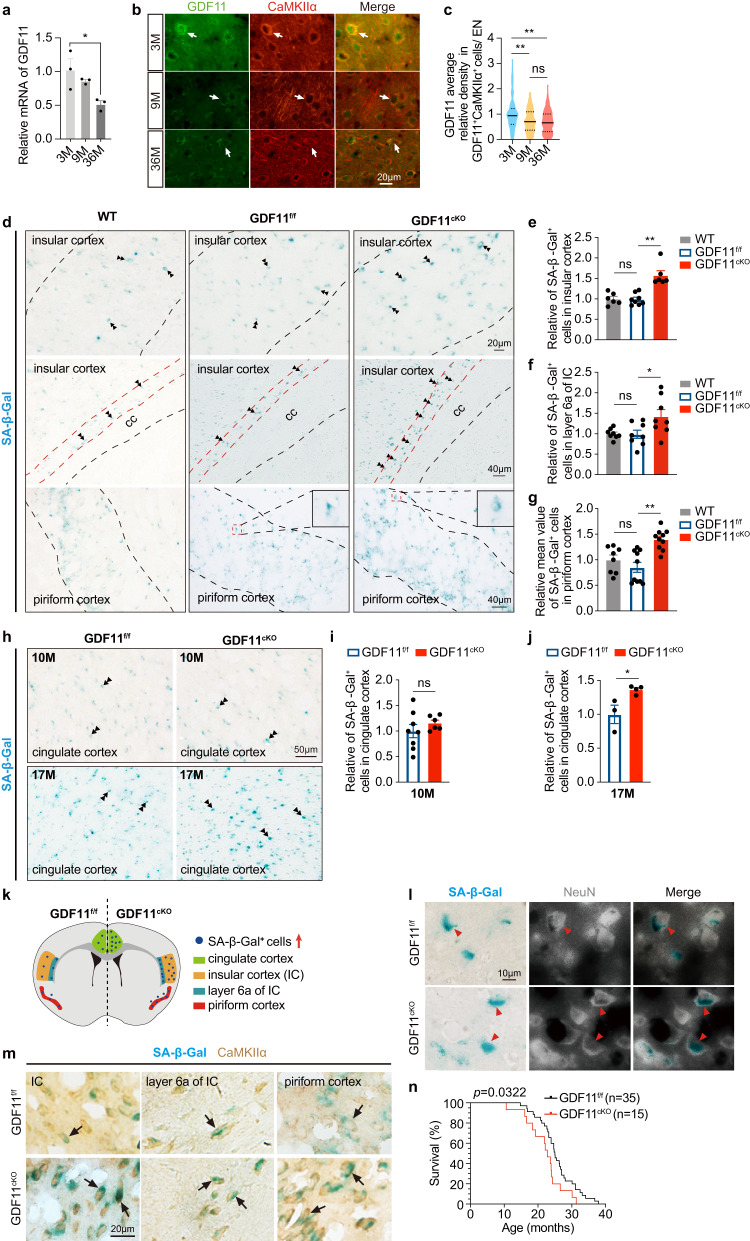
Selective deletion of GDF11 in the EN of the CNS accelerates their own senescence preferentially in the insular, piriform and cingulate cortices and shortens lifespan in mice. **a** Quantification by qPCR of the relative mRNA of GDF11 in the brain of the WT mice aged 3 M, 9 M or 36 M (*n* = 3 mice/group). **b** Immunofluorescence double labelling of GDF11 (green) and CaMKIIα (red) in the cerebral cortices of the mice aged 3 M, 9 M and 36 M. One GDF11^+^CaMKIIα^+^ neuron is indicated by an arrow as an example per group. **c** Quantification of the average gray value of GDF11 in GDF11^+^CaMKIIα^+^ neurons in the cerebral cortices of the mice aged 3 M, 9 M and 36 M (3 M, *n* = 140; 9 M, *n* = 160; 36 M, *n* = 232 cells). **d**–**g** Representative images (**d**) and quantification (**e**–**g**) of the SA-β-Gal^+^ cells in layers 4 and 5 (**d**, up, and **e**, the dashed lines indicate the borders of layers 4 and 5, WT, *n* = 6; GDF11^f/f^, *n* = 8; GDF11^cKO^, *n* = 6), layer 6a (**d**, middle, and **f** layer 6a is the deep layer cortex near the corpus callosum (CC), WT, *n* = 8; GDF11^f/f^, n = 8; GDF11^cKO^, *n* = 8) of the insular cortex (IC), and layers 2 and 3 of the piriform cortex (**d**, down, and **g** the dashed lines indicate the borders of layers 2 and 3, WT, *n* = 8; GDF11^f/f^, *n* = 10; GDF11^cKO^, *n* = 10) of GDF11^cKO^ or GDF11^f/f^ or WT mice aged 10 M. **h–j** Representative images (**h**) and quantification of the SA-β-Gal^+^ cells in the cingulate cortex of GDF11^cKO^ or GDF11^f/f^ mice aged 10 M (**i**, GDF11^f/f^, *n* = 8; GDF11^cKO^, *n* = 6) and 17 M (**j**, GDF11^f/f^, *n* = 3; GDF11^cKO^, *n* = 4). Examples of the SA-β-Gal^+^ cells are indicated by double arrowheads in (**d**, **h**). **k** A schematic summary on the distribution of the SA-β-Gal^+^ cells in the brain of GDF11^cKO^ or GDF11^f/f^ mice aged 10 M and 17 M. **l** Representative images of double labelling of SA-β-Gal staining (blue) and immunofluorescence of NeuN (fluorescence shown in white) in the insular cortex of GDF11^cKO^ or GDF11^f/f^ mice aged 10 M. Examples of the SA-β-Gal^+^NeuN^+^ neurons are indicated by red arrowheads. **m** Representative images of double labelling of SA-β-Gal staining (blue) and immunohistochemical staining of CaMKIIα (brown) in the cerebral cortices of GDF11^cKO^ or GDF11^f/f^ mice aged 10 M. Examples of the SA-β-Gal^+^CaMKIIα^+^ ENs are indicated by black arrows. **n** Survival curves of GDF11^f/f^ (*n* = 35 mice) and GDF11^cKO^ mice (*n* = 15 mice) which died naturally, and log-rank test *P* value was shown. Median survival is 25 months in GDF11^f/f^ mice and 22.8 months in GDF11^cKO^ mice. Scale bars, as shown on the images, 20 μm (**b**, **d** up, **m**), 40 μm (**d**, middle and down), 50 μm (**h**) and 10 μm (**l**). Data are presented as mean ± SEM. *P < 0.05, **P < 0.01. **a** (*F* (2, 6) = 6.672, **e** 0.0298; 3 M versus 36 M, *P* = 0.0270), **c** (*F* (2529) = 18.77, *P* < 0.0001; 3 M versus 9 M, *P* < 0.0001; 3 M versus 36 M, *P* < 0.0001; 9 M versus 36 M, *P* = 0.5477), **e** (*F* (2, 17) = 20.14, *P* < 0.0001; WT versus GDF11^f/f^, *P* = 0.9950; GDF11^f/f^, versus GDF11^cKO^, *P* < 0.0001), **f** (*F* (2, 21) = 4.825, *P* = 0.0189; WT versus GDF11^f/f^, *P* = 0.9963; GDF11^f/f^, versus GDF11^cKO^, *P* = 0.0322) and **g** (*F* (2, 25) = 11.61, *P* = 0.0003; WT versus GDF11^f/^f, *P* = 0.4738; GDF11^f/f^, versus GDF11^cKO^, *P* = 0.0002). One-way ANOVA with post Tukey multiple comparisons test. **i** (*P* = 0.3427) and **j** (*P* = 0.0280), unpaired two-tailed *t* test. Source data are provided with this paper.

To explore whether the transcription of GDF11 changes with progressive ageing, we found that the mRNA of GDF11 in the whole brain (from olfactory bulb to medulla oblongata) of the mouse halved at age of 36 M while no significant reduction at 9 M in comparison with 3 M (Fig. 2a). Immunofluorescence double labelling intensity analysis further showed that the protein level of GDF11 in the EN of the mouse cerebral cortex exhibited a reduction with chronological ageing at age of both 9 M and 36 M when compared with 3 M (Fig. 2b, c). These results arouse our curiosity on the role of endogenous GDF11 during brain ageing. To delete GDF11 in vivo, we created a new mouse line with a Loxp-flanked (floxp) allele of exon 2 of GDF11 (GDF11^f/f^) (Supplementary Fig. 3a, b) to avoid the embryonic lethality caused by systemic deletion of GDF11 in GDF11^−/−^ mouse^18^. Through GDF11^f/f^ mouse crossing with CaMKIIα-Cre mouse, in which Cre/Loxp recombination has been confirmed to occur during the third postnatal week^19^, we selectively deleted GDF11 in the EN of the mouse CNS (GDF11^cKO^, Supplementary Fig. 3a, b). Our PCR results verified that GDF11 gene was indeed deleted in GDF11^cKO^ mice (Supplementary Fig. 3c).

To explore whether selective deletion of GDF11 in the EN of the CNS affects neuronal senescence, we performed senescence-associated β-galactosidase staining (SA-β-Gal staining), a widely used method to detect senescent cells^20^. Quantification of SA-β-Gal^+^ senescent cells showed that at age of 10 M, no difference was seen between WT mouse and the GDF11^f/f^ mouse, suggesting that insertion of floxp gene did not affect cortical cellular senescence in mice. At age of 10 M, when compared with WT mouse and the GDF11^f/f^ mouse, an increase in SA-β-Gal^+^ senescent cells was observed exclusively in the following, but not other, brain regions of the GDF11^cKO^ mouse (Fig. 2d–g). In layers 4 and 5 (top, Fig. 2d, e) and layer 6a of the insular cortex (IC), and the layer 6a in IC closely surrounds the external capsule end of the corpus callosum (middle, Fig. 2d, f), and in layers 2 (pyramidal layer) and 3 (polymorph layer) of the piriform cortices of the GDF11^cKO^ mouse, over 50% more SA-β-Gal^+^ cells were found (bottom, Fig. 2d, g). Interestingly, at age of 17 M, in addition to the IC and the piriform cortex, an increase of SA-β-Gal^+^ senescent cells was also seen in the cingulate cortex of mouse albeit no difference was seen at age of 10 M (Fig. 2h–j). These results indicate that loss of GDF11 in the EN of CNS induces cellular senescence preferentially in certain cortical regions such as the insular, piriform and cingulate cortices (summarized in Fig. 2k).

To clarify which cell type gets senescent, sequential labelling of SA-β-Gal staining and immunofluorescence or immunohistochemistry was performed. We found that the predominant SA-β-Gal^+^ cells were double labeled with a pan-neuronal marker NeuN (Fig. 2l) and an EN marker CaMKIIα (Fig. 2m) in the cortices of the GDF11^f/f^ and GDF11^cKO^ mice aged 10 M, with more SA-β-Gal^+^ cells in GDF11^cKO^ mice than GDF11^f/f^ mice. Our results indicate that specific knocking-out GDF11 in the EN accelerates EN senescence preferentially in the insular, piriform and cingulate cortices (Fig. 2k), demonstrating that the endogenous GDF11 in the EN is required for them to maintain themselves in young status.

A recent study reported that the activity of the EN in the brain increased with ageing while inhibition of the EN activity extends lifespan in *C. elegans*^11^. However, so far it remains unknown whether the EN regulates lifespan in species other than *C. elegans*. To this end, we specifically deleted GDF11 in the EN of the mouse CNS, and found that the median survival rate was 25 months for GDF11^f/f^ mice while 22.8 months for GDF11^cKO^ mice (Fig. 2n), equivalent to a 10% reduction in lifespan for GDF11^cKO^ mice. This result indicates that loss of GDF11 in the EN shortens lifespan in mice, highlighting the essential role of GDF11 in the EN on not only brain ageing but also organismal ageing. These results reveal the first molecular mechanism underlying the EN senescence in vivo.

### Loss of GDF11 induces cellular senescence in vitro

**Fig. 3:**
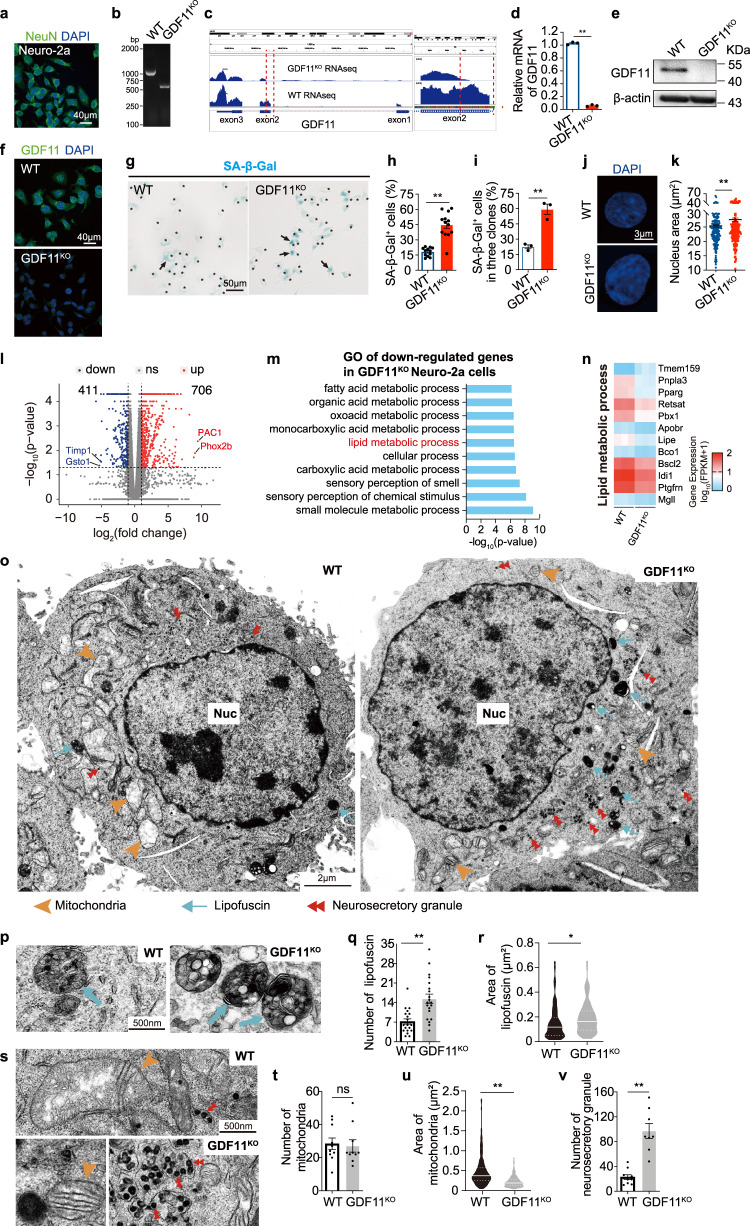
In vitro loss of GDF11 induces cellular senescence phenotypes and senescence-associated transcriptional programs. **a** Immunofluorescence image of NeuN (green) in Neuro-2a cells (*n* = 6 fields). Scale bar, 40 μm. **b** PCR of the cell genomes verified successful knockout of the targeted part of exon 2 of GDF11 in Neuro-2a cells (GDF11^KO^) (*n* = 3 clones of GDF11^KO^ cells). **c** Verification of GDF11 knockout by comparing the mRNA enrichment tracks of GDF11 between GDF11^KO^ and WT Neuro2a cells by bulk RNA-seq. **d** Quantification of the relative mRNA of GDF11 in the GDF11^KO^ and WT Neuro-2a cells by qPCR (*n* = 3 biological repeats/group). **e**, **f** Western blot (**e**) and Immunofluorescence of GDF11 (**f**, scale bar, 40 μm) in GDF11^KO^ or WT Neuro-2a cells (*n* = 3 biological repeats/ group). **g**, **h** Representative images (**g**) and quantification (**h**, GDF11^KO^, *n* = 13; WT, *n* = 12 fields) of the SA-β-Gal^+^ cells (blue) in GDF11^KO^ and WT Neuro-2a cells. All cells are indicated by black stars, and a few representative SA-β-Gal^+^ cells are indicated by black arrows. Scale bar, 50 μm. **i** Quantification of SA-β-Gal^+^ cells in 3 independent clones of GDF11^KO^ and WT Neuro-2a cells (GDF11^KO^, *n* = 3; WT, *n* = 3 clones). **j**, **k** Representative images (**j**, DAPI, blue) and quantification (**k**, GDF11^KO^, *n* = 234 cells; WT, *n* = 211 cells) of the nuclei of GDF11^KO^ and WT Neuro-2a cells. Scale bar, 3 μm. **l** Volcano plot of upregulated (706) and downregulated (411) genes caused by deletion of GDF11 in Neuro-2a cells and revealed by bulk-RNA-seq (*n* = 3 clones). **m** Bulk RNA-seq gene ontology (GO) analysis reveals the top 10 enriched biological processes downregulated by GDF11 deletion in Neuro-2a cells, and the logarithm base 2 of the fold change below −1 was included. **n** Heatmap of downregulated (11) or upregulated (1) genes involved in “lipid metabolic process” listed in m or “lipid droplets” caused by deletion of GDF11 in Neuro-2a cells, and the logarithm base 2 of the fold change above 1 or below −1 was included. **o** Representative images of transmission electron microscope (TEM) show the ultrastructure features of GDF11^KO^ and WT Neuro-2a cells. Cell nucleus (Nuc), lipofuscin (light blue arrows), neurosecretory granules (red double arrowheads) and mitochondrion (brown arrowheads) are indicated as examples. Scale bars, 2 μm. **p**–**r** Representative TEM images (**p**, lipofuscins, light blue arrows) and quantification of the number (Q, GDF11^KO^, *n* = 20 cells; WT, *n* = 20 cells) or the area (**r**, GDF11^KO^, *n* = 141; WT, *n* = 85 lipofuscins) of lipofuscins in the GDF11^KO^ and WT Neuro-2a cells. Scale bars, 500 nm. **s**–**u** Representative TEM images (**s**, mitochondrion, brown arrowheads; neurosecretory granules, red double arrowheads) and quantification of the number (**t**, GDF11^KO^, *n* = 10 cells; WT, *n* = 10 cells) or the area (**u**, GDF11^KO^, *n* = 299; WT, *n* = 254 mitochondria) of the mitochondria of the GDF11^KO^ and WT Neuro-2a cells. Scale bars, 500 nm. **v** Quantification of the number of neurosecretory granules (GDF11^KO^, *n* = 8 cells; WT, *n* = 10 cells) of the GDF11^KO^ and WT Neuro-2a cells. Data are presented as mean ± SEM. *P < 0.05, **P < 0.01 and “ns” indicates not significant, **d** (*P* < 0.0001), **h** (*P* < 0.0001), **i** (*P* = 0.0024), **k** (*P* = 0.0030), **q** (*P* = 0.0002), **r** (*P* = 0.0274), **t** (*P* = 0.8009), **u** (*P* < 0.0001), **v** (*P* = 0.0047), unpaired two-tailed *t* test. Source data are provided with this paper.

To directly assess whether loss of GDF11 in neuronal cells affects cellular senescence, we adopted CRISPR/Cas9 gene knockout strategy and specifically deleted part of exon 2 of *GDF11* gene in Neuro-2a cells (Supplementary Fig. 4a, b) that expressed NeuN (Fig. 3a), consistent with their identity of a neuronal cell line. The GDF11^KO^ or WT Neuro-2a cells were expanded and confirmed through single clone expansion. GDF11 deletion was confirmed at the DNA, mRNA and protein level. Our PCR results yielded 992 bp for WT and 514 bp for GDF11^KO^ Neuro-2a cells (Fig. 3b), and RNA sequencing (RNA-seq) showed the precise deletion of the targeted part of exon 2 of *GDF11* (Fig. 3c), and the deletion was also verified by qPCR (Fig. 3d) and the abolished labeling of GDF11 in western blot (Fig. 3e) and immunofluorescence (Fig. 3f), verifying a bona-fide deletion of GDF11 exon 2 in GDF11^KO^ cells. 3 independent clones of GDF11^KO^ cells were obtained, and cultured continuously up to 65 days in vitro (DIV), equivalent to 21 passages or roughly 42 cell divisions to collect sufficient number of cells for the planned experiments. Similar to our in vivo results, knocking-out GDF11 caused an over twofold increase in the proportion of SA-β-Gal^+^ cells (Fig. 3g, h), and consistent results were obtained in all three independent clones (Fig. 3i and Supplementary Fig. 4c–f), showing that deleting GDF11 indeed exacerbated cellular senescence in vitro. Loss of GDF11-induced increase of SA-β-Gal^+^ cells was also confirmed at 20 DIV, equivalent to 6 passages or 12 cell divisions. Our results that loss of GDF11 caused an increase of SA-β-Gal^+^ cells both in vivo and in vitro suggest an autophagy/mitophagy dysfunction, manifesting a feature of post-mitotic neuronal senescence^21^. By measuring the nucleus area of the microscopic images, we found that an increase in nucleus area in cells lacking GDF11 in comparison with WT cells (Fig. 3j, k), manifesting the enlarged nucleus size, another known feature of cellular senescence^22^. Our data indicate that GDF11 deletion exacerbates cellular senescence and enlarges cell nuclear size on average.

To examine the global transcriptional alterations caused by GDF11 deletion, we performed bulk RNA-seq of GDF11^KO^ and WT Neuro-2a cells. In comparison with WT cells, 706 genes were upregulated while 411 genes were downregulated in GDF11^KO^ cells (Fig. 3l). Among the downregulated genes, glutathione S-transferase omega 1 (*Gsto1*) was in the top list (Fig. 3l), consistent with previous reports that the level of Gsto1 is associated with delaying age-at-onset of Alzheimer disease (AD) and Parkinson disease (PD) in patients^23^, and mutation at rs4925 of *Gsto1* is a genetic marker for mild cognitive impairment in late-onset AD^24^. We found that the most upregulated gene was *Adcyap1r1*, adenylate cyclase-activating polypeptide 1 receptor 1 (Pac1 receptor) (Fig. 3l), which was upregulated in the brain of aged rat in a previous study^25^.

To understand the general biological pathways upregulated or downregulated by GDF11 deletion, we did gene ontology (GO) analysis. Among the 20 enriched cellular senescence and ageing-related biological processes, loss of GDF11 enhanced biological processes which are mainly relevant to cell adhesion, cell proliferation, response to cytokine stimulus, synapse and learning or memory (Supplementary Fig. 4g), suggesting that loss of GDF11 affects a spectrum of senescence-associated biological processes. Among the downregulated biological processes, 7 out of the top 10 enriched pathways were relevant to metabolism, associating GDF11 with metabolism. These results are consistent with cellular senescence profiles^8^. Among all enriched pathways, lipid metabolic process ranked the sixth (Fig. 3m). We further selected the genes up or downregulated over twofold in lipid metabolic process and presented in the heatmap (Fig. 3n), and found that Tmem159 (also known as lipid droplet assembly factor 1, LDAF1) was upregulated whereas Pnpla3 (Adiponutrin) was downregulated by GDF11 deletion (Fig. 3n). Our western blot results validated that loss of GDF11 upregulated Tmem159 to fivefolds at protein level (Supplementary Fig. 5a, b). Consistently, previous studies showed that Tmem159 (LDAF1) plays a pivotal role in lipid droplet formation^26^ whereas Pnpla3 participates in restoration of lipid upon aberrant lipid accumulation^27^. These data suggest that GDF11 deletion might cause lipid droplets accumulation. To test this possibility, we studied the cellular ultrastructure under transmission electron microscope (TEM). We found that in comparison with WT cells (Fig. 3o), loss of GDF11 doubled the number (Fig. 3p, q) and increased the area (Fig. 3o–r) of lipofuscin, manifesting lipofuscin accumulation, a typical feature of senescent cells^28^. In WT cells the normal lipofuscin (secondary lysosome) was smaller and contained smaller electron dense particles (Fig. 3p), whereas in GDF11^KO^ cells there were more and larger lipofuscin that contained many larger electron dense particles, onion-like layered structures and more lipid droplets (Fig. 3p). In addition, loss of GDF11 did not affect the number of mitochondria (Fig. 3s, t) but caused a decrease in mitochondrial area (Fig. 3s, u), suggesting GDF11 deletion might impair mitochondria. Together with the results that loss of GDF11 led to lipofuscin accumulation, these results support that loss of GDF11 may induce senescence-associated mitochondria dysfunction, a feature of post-mitotic neuronal senescence^21^. Besides, in GDF11^KO^ cells, the number of neurosecretory granules increased to five times of WT cells (Fig. 3s, v), suggesting that loss of GDF11 may affect neurosecretory process which could involve production, release or degradation of neuronal substances. Together, GDF11 deletion increases SA-β-Gal^+^ cells, likely impair lipid metabolism, causes accumulation of lipofuscin and neurosecretory granules in the cytoplasm of the cells, and may impair mitochondria, manifesting features of post-mitotic neuronal senescence^21,29^.

### Loss of GDF11 in excitatory neurons impairs cognition and memory

**Fig. 4:**
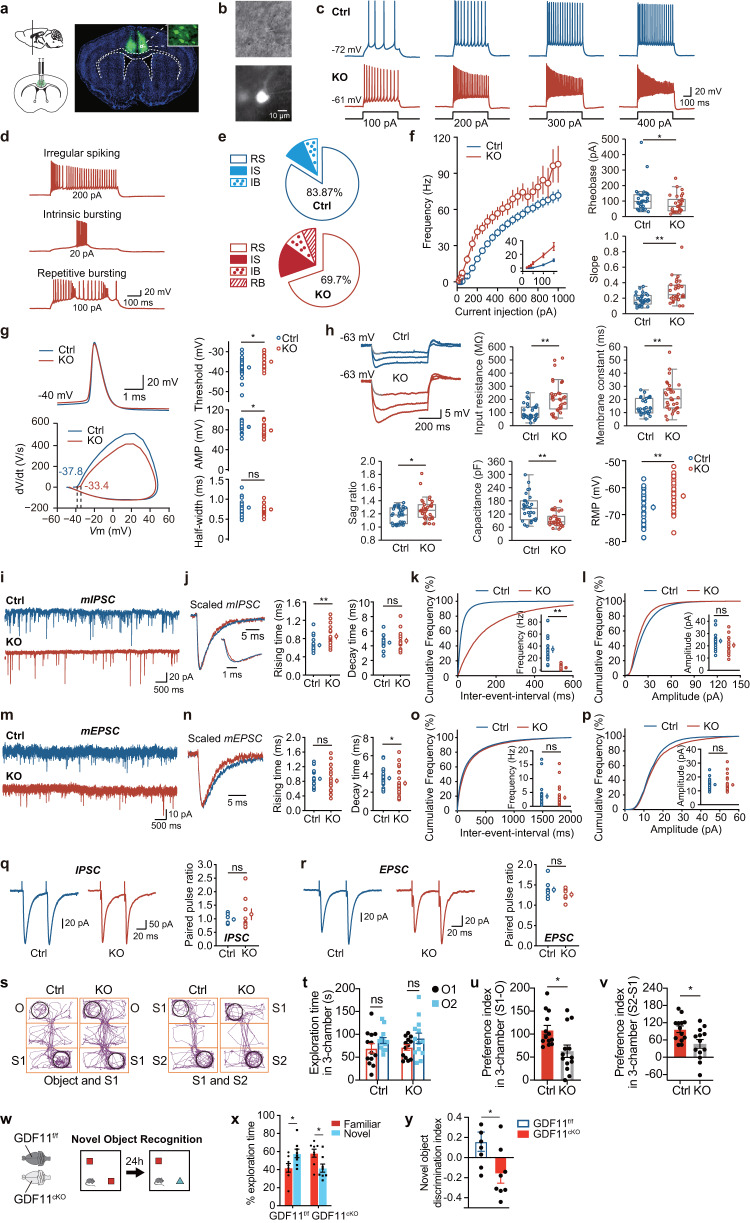
Selective deletion of GDF11 in the EN causes their own hyperexcitability and deteriorates social cognition and object recognition memory in mice. **a** Schematic diagrams (left) and representative images (right) of the cingulate gyrus 2 (Cg2), in the prefrontal cortex of GDF11^f/f^ mice aged 4M-5M, where bilateral focal injection of AAV9-CaMKIIα-Cre-P2A-GFP virus (KO) or AAV9-CaMKIIα-GFP virus (Ctrl) was received at age of 2–3 M and survived for two more months. **b** Infrared-differential interference contrast (IR-DIC) image (top) and GFP fluorescent image (bottom) of an example of GFP^+^ EN which is undergoing whole-cell patch clamp recording (*n* = 64 cells from six mice). **c** Representative whole-cell recordings in brain slice of a control EN (in Cg2 of GDF11^f/f^ mice, Ctrl, blue) and a GDF11 deleted-EN (in Cg2 of fGDF11^cKO^ mice, KO, red) show the firing of action potentials (AP) in response to a series of step current injections. **d** Examples show typical firing patterns of GFP^+^ EN of fGDF11^cKO^ mice. **e** Pie graphs show the percentage of GFP^+^ EN with diverse firing patterns (RS, regular spiking; IS, irregular spiking; IB, intrinsic bursting; RB, repetitive bursting) in WT or KO mice. **f** Left, plots of the AP frequency as a function of injected currents. Curves are color coded (Ctrl, blue, *n* = 31 cells from three mice; KO, red, *n* = 33 cells from three mice). Inset shows the beginning of the curve. Right, plots of the rheobase (Ctrl: 113 ± 16 vs. KO: 81 ± 10 pA, *P* = 0.049) and slope (Ctrl: 0.18 ± 0.01 vs. KO: 0.30 ± 0.03, *P* = 0.000) in the two groups (Ctrl, *n* = 31 cells from three mice; KO, *n* = 30 cells from three mice). **g** Left, representative AP waveforms (top) and phase plots (bottom) from Ctrl (blue) or KO (red) group. Right, plots of the AP threshold (Ctrl: −37.9 ± 0.8 vs. KO: −35.0 ± 0.7 mV, *P* = 0.014), amplitude (AMP) (Ctrl: 85.8 ± 1.6 vs. KO: 78.6 ± 2.2 mV, *P* = 0.010) and half-width (Ctrl: 0.79 ± 0.03 vs. KO: 0.74 ± 0.03 ms, *P* = 0.30) in the two groups (Ctrl, *n* = 29 cells from three mice; KO, *n* = 24 cells from three mice). **h** Left-top, representative membrane potential responses to negative current pulses from Ctrl (blue) or KO (red) groups. Plots of the input resistance (Ctrl: 104 ± 10 vs. KO: 214 ± 21 MΩ, *P* = 0.000), membrane constant (Ctrl: 14.4 ± 1.1 vs. KO: 22.1 ± 2.0 ms, *P* = 0.003), Sag ratio (Ctrl: 1.18 ± 0.02 vs. KO: 1.27 ± 0.03, *P* = 0.033), membrane capacitance (Ctrl: 147 ± 11 vs. KO: 95 ± 5 pF, *P* = 0.000) and RMP (Ctrl: −67.3 ± 1.0 vs. KO: −63.1 ± 0.9 mV, *P* = 0.004) in the two groups (Ctrl, *n* = 31 cells from three mice; KO, *n* = 33 cells from three mice). **i** Representative whole-cell recordings of mIPSC from the EN in GDF11^f/f^ mice (Ctrl, blue) and fGDF11^cKO^ mice (KO, red). **j** Left, scaled mIPSC examples in the two groups. Right, plots of rising time (Ctrl: 0.65 ± 0.04 vs. KO: 0.85 ± 0.06 ms, *P* = 0.005) and decay time (Ctrl: 4.44 ± 0.21 vs. KO: 4.69 ± 0.34 ms, *P* = 0.53) of mIPSCs in the two groups (Ctrl, *n* = 18 cells from four mice; KO, *n* = 16 cells from four mice). **k**, **l** Cumulative frequency curve of the inter-event-interval (**k**) and amplitude (**l**) of mIPSCs. Insets show the group plots of mIPSC frequency (**k**, Ctrl: 34.6 ± 5.2 vs. KO: 4.0 ± 0.9 Hz, *P* = 0.000) and amplitude (**l**, Ctrl: 24.0 ± 1.6 vs. KO: 20.5 ± 1.8 pA, *P* = 0.16). **m**–**p** Recordings of mEPSCs (Ctrl, *n* = 24 cells from four mice; KO, n = 28 cells from 4 mice) and similar plots as the mIPSCs shown above. Rising time (**n**, ctrl: 0.87 ± 0.05 vs. KO: 0.81 ± 0.06 ms, *P* = 0.46); Decay time (**n**, ctrl: 3.54 ± 0.20 vs. KO: 2.98 ± 0.24 ms, *P* = 0.041); Frequency (**o**, Ctrl: 3.66 ± 0.84 vs. KO: 3.13 ± 0.65 Hz, p = 0.82); Amplitude (**p**, Ctrl: 14.5 ± 0.8 vs. KO: 14.3 ± 0.9 pA, *P* = 0.33). **q**, **r** Representative traces showing IPSC (**q**, left) or EPSC (**r**, left) evoked by extracellular electric stimulations for the comparison of paired-pulse ratio (PPR) in GDF11^f/f^ mice (Ctrl, blue) and fGDF11^cKO^ mice (KO, red). Group plots of PPR for IPSC (**q**, right, Ctrl, *n* = 7 cells from 3 mice: 0.98 ± 0.07 vs. KO, *n* = 9 cells from three mice: 1.16 ± 0.20, *P* = 0.92) and EPSC (**r**, right, Ctrl, *n* = 9 cells from 3 mice: 1.38 ± 0.07 vs. KO, *n* = 6 cells from three mice: 1.26 ± 0.06, *P* = 0.24). **s** Track diagrams in the 3-chamber test (3CT) between the fGDF11^cKO^ (KO) and GDF11^f/f^ (Ctrl) mice aged 4–5 M. O object, S1 stranger mouse, S2 new stranger mouse. **t** Quantification of the exploration time in 3CT (KO, *n* = 13; Ctrl, *n* = 13 mice) on objects between the fGDF11^cKO^ (KO) and GDF11^f/f^ (Ctrl) mice aged 4–5 M. O1, object 1; O2, object 2. **u** Quantification of the preference index (S1-O) between the S1 and object in the KO and Ctrl groups (KO, *n* = 13; Ctrl, *n* = 13 mice). **v** Quantification of the preference index (S2-S1) between the S2 and S1 in the KO and Ctrl groups (KO, *n* = 13; Ctrl, *n* = 13 mice). **w** Schematic diagram of the novel object recognition test (NORT) between the GDF11^cKO^ and GDF11^f/f^ mice aged 10 M. Red squares indicate the familiar toy while blue triangle indicates a novel toy. **x** Quantification of the percentage of exploration time (GDF11^cKO^, *n* = 9; GDF11^f/f^, *n* = 6 mice) on the familiar or a novel toy in the GDF11^cKO^ and GDF11^f/f^ mice aged 10 M. **y** Quantification of the novel object discrimination index ((novel-familiar)/(novel + familiar)) between the familiar or a novel toy in the GDF11^cKO^ and GDF11^f/f^ mice aged 10 M (GDF11^cKO^, *n* = 9; GDF11^f/f^, *n* = 6 mice). Data are presented as mean ± SEM. Whisker boxplots in (**f**, **h**) represent the median and interquartile range; whiskers represent 1.5× interquartile range. *P < 0.05, **P < 0.01 and “ns” represents not significant. **f** (Rheobase/Slope), **h** (Input resistance/Membrane constant/Sag ratio/Capacitance), **j** (Rising time), **k**, **n** (Decay time), **o**–**q** Mann–Whitney *U* test. **g**, **h** (RMP), **j** (Decay time), **l**, **n** (Rising time), **r**, **u** (*P* = 0.0118), **v** (*P* = 0.0128), **x** (GDF11^f/f^: Familiar versus Novel, *P* = 0.0331; GDF11^cKO^: Familiar versus Novel, *P* = 0.0188) and **y** (*P* = 0.0254), unpaired two-tailed *t* test. **t** (Ctrl: O1 versus O2, *P* = 0.3210; KO: O1 versus O2, *P* = 0.2200), two-way ANOVA with post Sidak’s multiple comparisons test. Source data are provided with this paper.

Having shown that loss of GDF11 in the EN causes EN senescence preferentially in three brain regions: the insular, piriform and cingulate cortices (Fig. 2). Among these three cortical regions, we chose to target the cingulate gyrus 2 (Cg2) in the prefrontal cortex because it is easier for accurate microinjection and it has been established a battery of specific behavioral tests on Cg^30,31^. To further explore whether selective loss of GDF11 in the EN affects their function, we took Cre/loxp strategy and exclusively deleted GDF11 in the EN by a focal injection of AAV9-CaMKIIa-Cre-EGFP into Cg2 of the GDF11^f/f^ mouse (fGDF11^cKO^, abbreviated to KO), while a focal injection of AAV9-CaMKIIα-EGFP virus was used as the control (Ctrl) (Fig. 4a). First, our sequential labelling of SA-β-Gal staining and immunohistochemistry of CaMKIIα confirmed that in vivo specific and focal knocking-out GDF11 in the EN in Cg2 promoted a focal excitatory neuronal senescence indeed at age of 4–5 M (Supplementary Fig. 6). Our whole-cell patch clamp recording revealed an increase in excitability of the EN with GDF11 deleted at age of 4–5 M (Fig. 4b–f): in response to injection of a series of current pulses, compared with the control, the EN with GDF11 deleted exhibited higher frequency of action potential (AP) (Fig. 4c–e). The pooled frequency-current curves (Fig. 4f) displayed a hyperexcitability in KO EN. Close examination of the AP waveform revealed a more depolarized voltage threshold (Fig. 4g) and a slightly smaller peak amplitude (AMP) in KO EN (Fig. 4g). Membrane potential responses to negative current pulses showed that KO EN possessed greater input resistance and membrane constant, resulting in smaller cell capacitance but bigger sag ratio (Fig. 4h). The elevated input resistance and resting membrane potential (RMP) (Fig. 4h) may overcome the small increase in voltage threshold, causing an enhancement of neuronal excitability. These results show that loss of GDF11 alters active and passive membrane properties in the EN, resulting in their own hyperexcitability.

To further explore whether selective loss of GDF11 in the EN in Cg2 of the prefrontal cortex in GDF11^f/f^ mice affects their functional synaptic connectivity, we recorded both miniature excitatory and inhibitory postsynaptic currents (mEPSC and mIPSC) on the EN. Compared with the control, the EN with GDF11 deleted showed impaired mIPSC. The mIPSCs exhibited a dramatic decrease in their frequency (Fig. 4i, k) and an increase in the rising time (Fig. 4j) while the amplitude remained similar (Fig. 4l). However, no change was detected in mEPSCs on either frequency or amplitude (Fig. 4m–p). Using TEM we quantified both the excitatory and inhibitory synapses in Cg2. Our results showed that in vivo selective deletion of GDF11 in the EN in Cg2 caused the number of inhibitory synapses per excitatory neuron body decreased over 50%, which can reduce the inhibitory input onto the EN and thus reduces the frequency of mIPSCs (Supplementary Fig. 11a, b). Meanwhile, the number of excitatory synapses decreased, but the excitatory input per postsynaptic membrane increased in Cg2, which could form a dynamic balance and did not lead to the modification of mEPSC frequency (Supplementary Fig. 11c–f). These data indicate that loss of GDF11 in the EN decreases their inhibitory input while increases their excitatory input. To examine whether neurotransmitter release probability of these synaptic inputs is altered by GDF11 deletion, we examined the paired-pulse ratio (PPR) with extracellular stimulation (Fig. 4q, r). We found that neither IPSCs (Fig. 4q) nor EPSCs (Fig. 4r) displayed a change in PPR, suggesting that the reduced mIPSC frequency is not attributable to alterations in presynaptic release probability but may result from a change in the number of functional inhibitory synapses. In these electrophysiological experiments, the GFP^-^ EN in control mice and KO mice, and the EN rescued by re-expressed GDF11 in GDF11^cKO^ mice showed no difference in all above-mentioned parameters. These results indicate that in vivo GDF11 deletion in the EN decreases the inhibitory input but increases the excitatory input onto the EN, and the dynamic balance ultimately leads to neuronal hyperexcitability, manifesting an emerging feature of the aged EN.

Having shown that focal and selective deletion of GDF11 in the EN of Cg2 causes their own senescence and hyperexcitability, we wonder whether such effects could translate into behavioral changes. To explore this, a battery of behavioral tests was performed in order. Using three chamber test (3CT)^32^, we found that both fGDF11^cKO^ and control mice, aged 4–5 M, did not show a preference between testing objects 1 and 2 at habituation session (Fig. 4s, t). Interestingly, compared with the control mice, fGDF11^cKO^ mice exhibited lower level of not only social activity on spending time between the social chamber (S1) and the object chamber (O) during the sociability session (Fig. 4s, u) but also social memory on discriminating between the familiar (S1) and the novel (S2) mouse chambers (Fig. 4s, v), indicating impeded sociability and social memory in fGDF11^cKO^ mice. In both fGDF11^cKO^ and control mice, similar levels of locomotion in open field test (OFT), anxiety-like behavior in elevated plus maze (EPM) test, aversive stimuli-driven memory in passive avoidance test (PAT, light–dark transition test) and despair behavior in tail suspension test (TST) were found (Supplementary Fig. 7). These results indicate that focal deletion of GDF11 in the EN of Cg2 deteriorates social cognition in mice.

Since in vivo systemic and selective deletion of GDF11 in the EN of the CNS accelerates EN senescence preferentially in the insular and piriform cortices at age of 10 M (Fig. 2), we wonder whether these impairments could also affect cognition. OFT, EPM, Novel object recognition test (NORT) and PAT were conducted successively. Interestingly, at age of 10 M, during NORT under our experimental parameters^33^, in contrast to GDF11^f/f^ mice who spent more time to explore the novel objects, GDF11^cKO^ mice failed to distinguish between the novel and the familiar objects (Fig. 4w–y), indicating a declined ability for GDF11^cKO^ mice to recognize new objects or environmental cues, manifesting the impeded object recognition memory in aged animals^33^. However, in comparison with GDF11^f/f^ mice, GDF11^cKO^ mice exhibited no difference on locomotion during OFT, anxiety level in EPM and aversive stimuli-driven memory in PAT (Supplementary Fig. 8). Collectively, both focal and systemic deletion of GDF11 in the EN deteriorates sociability, social memory and object recognition memory in mice, indicating that induction of cellular senescence in the EN by deleting GDF11 is sufficient to cause cognitive decline.

### GDF11 deletion shapes an excitatory neuronal senescence profile

**Fig. 5:**
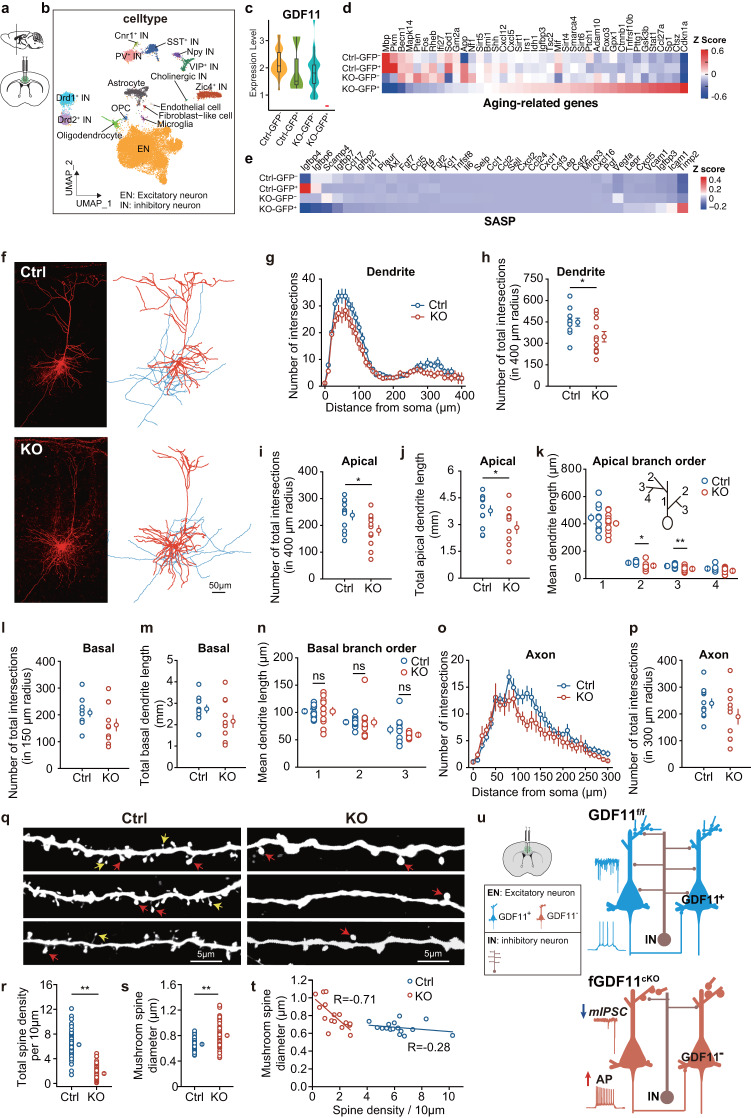
In vivo selective deletion of GDF11 in the EN drives transcriptional programs associated with brain ageing and prunes and shortens their apical dendrites. **a** Schematic diagrams of the cingulate gyrus 2 (Cg2), in the prefrontal cortex of GDF11^f/f^ mice aged 4–5 M, where bilateral focal injection of AAV9-CaMKIIα-Cre-P2A-GFP virus (KO) or AAV9-CaMKIIα-GFP virus (Ctrl) was received at age of 2–3 M and survived for two more months. **b** UMAP of the clustered 16 cell types in snRNA-seq of the Cg2 in both 3 KO mice and 3 control mice (Ctrl) aged 4–5 M. **c** Violin chart of the relative mRNA of GDF11 by snRNA-seq in KO-GFP^+^, KO-GFP^-^, Ctrl-GFP^+^ or Ctrl-GFP^-^ EN. The KO-EN were divided into KO-GFP^+^ and KO-GFP^-^ groups whereas “Ctrl-EN” were divided into Ctrl-GFP^+^ and Ctrl-GFP^−^ groups. **d** and **e**, Heatmap shows the average transcription of downregulated and upregulated ageing-related genes (**d**) and SASP-related genes (**e**) in snRNA-seq of KO-GFP^+^, KO-GFP^−^, Ctrl-GFP^+^ or Ctrl-GFP^−^ EN. **f** Confocal images (Left) and 3D-reconstruction (Right) of representative EN from Ctrl (Top) or KO (Bottom) groups. Dendrites and soma are presented in red, and axons are in blue. Scale bar, 50 μm. **g**, **h** Plots of the number of intersections of dendrites (**g**) in the two groups (Ctrl, *n* = 11 cells from three mice; KO, *n* = 11 cells from three mice) and the group data showing the number of total dendrite intersections (**h**, Ctrl: 448 ± 28 vs. KO: 346 ± 36, *P* = 0.028). **i**–**k** Group data show the total number of apical dendrite intersections (**i**, Ctrl: 238 ± 17 vs. KO: 181 ± 18, *P* = 0.036), the total length of apical dendrites (**j**, Ctrl: 3.77 ± 0.28 vs. KO: 2.83 ± 0.34 mm, *P* = 0.044), and the apical branch orders against the averaged dendrite length (**k**, branch order 1, Ctrl: 445 ± 28 vs. KO: 403 ± 22 μm, *P* = 0.26; branch order 2, Ctrl: 115 ± 3 vs. KO: 93 ± 8 μm, *P* = 0.017; order 3, Ctrl: 91 ± 4 vs. KO: 70 ± 6 μm, *P* = 0.007; branch order 4, Ctrl: 72 ± 6 vs. KO: 56 ± 7 μm, *P* = 0.12) in the two groups (Ctrl, *n* = 11 cells from three mice; KO, *n* = 11 cells from three mice). **l**–**n** Group data comparing the number of total basal intersections (**l**, Ctrl: 207 ± 15 vs. KO: 162 ± 22, *P* = 0.11), total basal dendrite length (**m**, Ctrl: 2.73 ± 0.18 vs. KO: 2.16 ± 0.29 mm, *P* = 0.11) and the basal branch orders against the averaged dendrite length (**n**, branch order 1, Ctrl: 102 ± 4 vs. KO: 102 ± 8 μm, *P* = 0.98; branch order 2, Ctrl: 82 ± 3 vs. KO: 82 ± 9 μm, *P* = 0.32; order 3, Ctrl: 69 ± 8 vs. KO: 59 ± 2 μm, *P* = 0.25) in the two groups (Ctrl, *n* = 11 cells from three mice; KO, *n* = 11 cells from three mice). **o**, **p** Plots of the axon distance from soma against the number of intersections (**o**) in the two groups (Ctrl, *n* = 11 cells from three mice; KO, *n* = 11 cells from three mice). Group data show the number of total axon branches intersections (**p**, Ctrl: 239 ± 17 vs. KO: 190 ± 28, *P* = 0.15). **q** Confocal examples of dendritic spines (red arrows indicate the big mushroom spines while yellow arrows point to small mushroom spines) in the two groups. Scale bar, 5 μm. **r**, **s** Group data show total spine density per 10 μm (**r**, Ctrl: 6.28 ± 0.23 vs. KO: 1.61 ± 0.13/10 μm, *P* = 0.000) and mushroom spine diameter (**s**, Ctrl: 0.66 ± 0.01 vs. KO: 0.80 ± 0.02 μm, *P* = 0.000) in two groups (Ctrl, *n* = 68 dendrites from 16 cells; KO, *n* = 70 dendrites from 16 cells). **t** Plots of spine density against the mushroom spine diameter in the two groups (Ctrl, *n* = 16 cells from three mice; KO, *n* = 16 cells from three mice). **u** A schematic summary: GDF11 deletion results in hyperexcitability of the EN as reflected by an enhancement in their firing frequency (due to increased input resistance and elevated RMP) and a decrease in mIPSC frequency. In addition, GDF11 deletion in the EN prunes and shortens their apical dendrites, reduces their dendritic mushroom spine density while enlarges its size. Data are presented as mean ± SEM. *P < 0.05, **P < 0.01. **h**, **i**, **j**, **k**, **l**, **m**, **n**, **p**, unpaired two-tailed *t* test; **r**, **s**, Mann–Whitney *U* test. Source data are provided with this paper.

To gain insight into changes in gene transcription in the EN in which GDF11 is deleted, we performed unbiased high-throughput single-nucleus RNA-seq (snRNA-seq) on cells in Cg2 in mouse prefrontal cortex. In fGDF11^cKO^ (abbreviated to KO) mice GDF11 was selectively deleted only in the GFP^+^ EN while in control GDF11^f/f^ (Ctrl) mice no gene was designed to be deleted. After filtering, we ultimately obtained the transcriptomes of 24,803 single nuclei derived from the Cg2 of 3 fGDF11^cKO^ mice and 3 GDF11^f/f^ mice (Fig. 5a). With uniform manifold approximation and projection (UMAP), we totally annotated 16 cell types in Cg2 with canonical marker genes (Fig. 5b and Supplementary Fig. 9). GDF11 mRNA was exclusively depleted in KO-GFP^+^ group but remained unchanged in both GDF11^f/f^ control (Ctrl-GFP^+^ and Ctrl-GFP^-^) and KO-GFP^-^ group (Fig. 5c), confirming that deletion of GDF11 is specific and only in the EN.

To investigate whether loss of GDF11 in the EN affects known ageing-related genes, we selected the ageing-related genes and validated with published studies. Our snRNA-seq revealed that loss of GDF11 in the EN caused a transcriptional increase in the gene sets (Cdkn1a/p21, Ctsz and Sp1 were the top 3) which have been reported upregulated during brain ageing^5^ and a transcriptional decrease in those genes which have been shown downregulated during brain ageing^34^ (Mbp, Pkm and Becn1 were the top three downregulated, Fig. 5d). To explore whether loss of GDF11 induced EN senescence negatively influences other cells through the SASP, analysis of our snRNA-seq data showed that selective deletion of GDF11 in the EN upregulated genes such Icam1, Timp2 while downregulated genes such as Igfbp4, Igfbp6 and Scamp4 (Fig. 5e), consistent with the profiles of senescence-associated secretome^8,9^. Thus, loss of GDF11 in the EN drives transcriptional programs consistently associated with the SASP.

To test whether the morphological properties of the EN were affected by knocking-out GDF11, we added biocytin to the internal solution of patch pipettes and performed post hoc streptavidin staining of the recorded EN. Sholl analysis (WT: *n* = 11 vs. KO: *n* = 11 cells; Fig. 5f and Supplementary Fig. 10) revealed that at age of 4–5 M, knocking-out GDF11 caused a decrease in the number of dendritic branches (Fig. 5g, h), the number of apical dendrite intersections (Fig. 5i) and the apical dendrite length (Fig. 5j). Specifically, the apical dendrite length in branch order 2 and order 3 (Fig. 5k) were shortened. Knocking-out GDF11 also slightly but non-significantly reduced the number of basal dendrite intersections (Fig. 5l), the basal dendrite length (Fig. 5m, n) as well as the number of axon intersections (Fig. 5o, p).

Next, we compared the dendritic spines of the EN at high magnification (WT: *n* = 16; KO: *n* = 16) and found that loss of GDF11 impeded their spine morphology in two ways. Firstly, knocking-out GDF11 decreased the spine density (Fig. 5q, r). Secondly, it surprisingly enlarged the spine head of the remained mushroom spines (Fig. 5q, s). Moreover, the mushroom spine diameter showed a negative correlation with the spine density in the GDF11 knocking-out group, however, it displayed no obvious correlation in the control group (Fig. 5t). Our TEM semi-quantification results revealed that loss of GDF11 in the EN decreases their inhibitory input but increases their excitatory input (Supplementary Fig. 11), consistent with our electrophysiological data (Fig. 4c–h).

In vivo selective deletion of GDF11 in the EN affects the transcription of both the excitatory and the inhibitory synapse assembly related genes (Supplementary Fig. 12). The most upregulated Abi3bp triggers cellular senescence through p21 pathway^35^, and Wnt7a is an inducer of beta-catenin-independent cellular senescence^36^. Interestingly, transcription of Srgap2 was also upregulated by loss of GDF11 (Supplementary Fig. 12a, b). Srgap2 encodes SLIT-ROBO Rho-GTPase-activating protein 2 (SRGAP2) which promotes spine maturation, limits spine length in the mouse neocortex^37^, and increases the density of longer spines, a feature characterizing pyramidal neurons in human neocortex^38^. These previous results consistently support our results that loss of GDF11 in the EN decreases the total spine density while increase mushroom spine diameter (Fig. 5) and accelerates EN senescence (Fig. 2).

Collectively, loss of GDF11 in the EN prunes and shortens their dendrites, reduces their dendritic mushroom spine density while enlarges their size, indicating that loss of GDF11 impairs the capacity of the EN likely through receiving less inhibitory but more excitatory synaptic inputs (Fig. 5u).

### GDF11 deletion upregulates p21

**Fig. 6:**
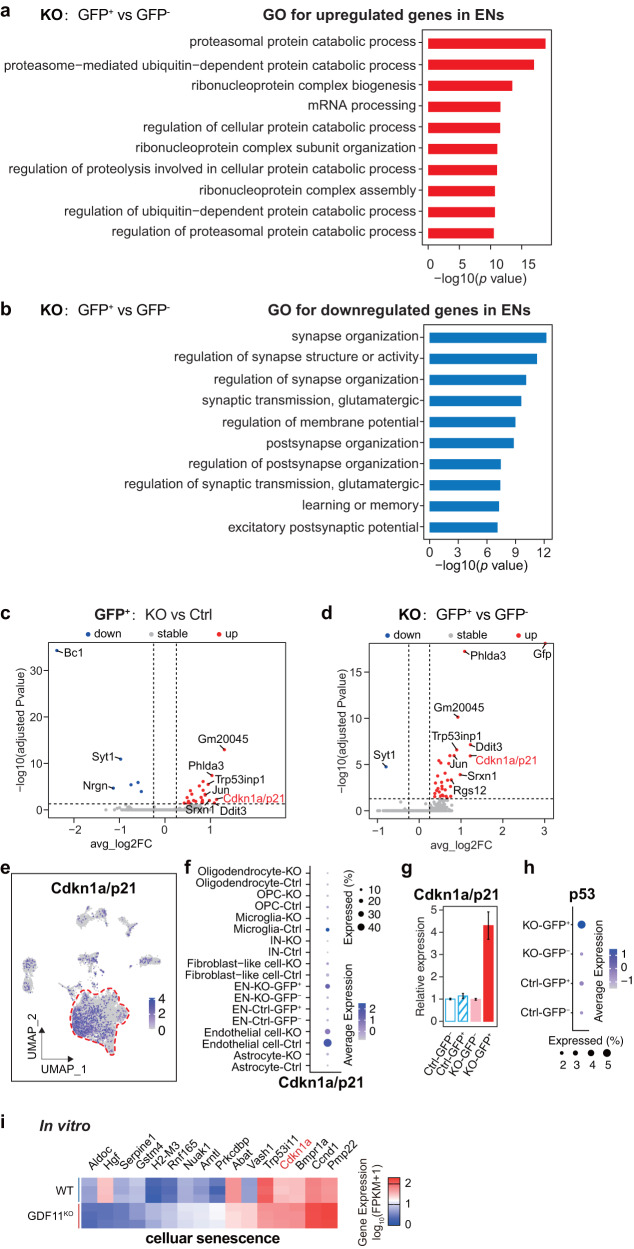
Loss of GDF11 upregulates p21 both in vivo and in vitro. **a**, **b** SnRNA-seq GO analysis reveals the top ten enriched biological processes of upregulated (**a**) or downregulated (**b**) in the KO-GFP^+^ EN in comparison with the KO-GFP^-^ EN, and the EN were obtained from the Cg2 of the “KO” mice and the “Ctrl” mice aged 4–5 M. **c** Volcano plot shows upregulated and downregulated DEGs in the KO-GFP^+^ EN in comparison with the Ctrl-GFP^+^ EN. Some of the top upregulated and downregulated genes were annotated. **c**, **d** FC fold change. *P* value was calculated using Wilcox test and adjusted for multiple testing using Benjamini–Hochberg correction. **d** Volcano plot shows upregulated and downregulated DEG in the KO-GFP^+^ EN in comparison with the KO-GFP^-^ EN. Some of the top upregulated and downregulated genes were indicated. **e** UMAP visualization highlights the distribution and the transcription of Cdkn1a/p21 in the identified cell types in snRNA-seq. **f** Dot plot representing the frequency and average transcription of Cdkn1a/p21 in the identified cell types in snRNA-seq. **g**, **h** Relative mRNA of Cdkn1a/p21 (**g**) or p53 (**h**) among four types of EN: Ctrl-GFP^-^, Ctrl-GFP^+^, KO-GFP^-^ and KO-GFP^+^ by snRNA-seq. **i** Heatmap of upregulated (10) and downregulated (6) genes involved in “cellular senescence” caused by deletion of GDF11 in Neuro-2a cells, and the logarithm base 2 of the fold change above 1 or below −1 was included. Data are presented as mean ± SEM. *P < 0.05, **P < 0.01 and “ns” indicates not significant. **a**, **b** Hypergeometric test with Benjamini and Hochberg (BH) correction. Source data are provided with this paper.

To investigate the cellular pathways which could mediate the accelerated EN senescence induced by deletion of GDF11 in the EN in vivo, gene set enrichment analysis (GSEA) on cellular pathways was performed. The same SnRNA-seq original data as used in Fig. 5 were also used here. Our GSEA analysis revealed that in comparison with the GFP^-^ EN, in vivo loss of GDF11 in the GFP^+^ EN upregulated 3, out of the top 10, pathways on ribonucleoprotein complex (Fig. 6a), consistent with previously reported upregulation of ribosome biogenesis pathway in neurons during natural ageing of mice^10^. In parallel, in vivo loss of GDF11 in the GFP^+^ EN downregulated 8, out of the top 10, pathways associated with synapse (Fig. 6b), consistent with both our current electrophysiological results (Figs. 4 and 5) and reported downregulation of synaptic transmission pathways in neurons during natural ageing of both mice^10^ and human^6,11^.

To further identify the transcriptional changes induced by in vivo loss of GDF11, we performed differential gene expression (DEG) analysis. Again, the same SnRNA-seq original data were used. DEGs of upregulated or downregulated genes yielded consistent results when KO was compared with two different controls, either between KO and control mice or in the same KO mouse: the GFP^+^ EN in KO-GFP^+^ versus Ctrl-GFP^+^ (Fig. 6c), and KO-GFP^+^ versus KO-GFP^-^ (Fig. 6d). In vivo loss of GDF11 in the EN downregulated synaptotagmin-1 (Syt1) (Fig. 6c, d) which has been shown to be involved in the docking, priming and fusion of synaptic vesicles^39^, consistent with the reduced neuronal synaptic transmission in the naturally aged brain of mice^10^ and human^6,11^.

Among the top upregulated genes, Cdkn1a/p21 was upregulated in GDF11 deleted EN (Fig. 6c–f). Interestingly, Cdkn1a/p21 has been shown as a pro-senescence factor for post-mitotic neurons^5,40^ and is upregulated in the naturally aged mouse brain^10^. Cell type distribution revealed that p21 was transcribed predominantly in the EN albeit also in other cell types with much smaller numbers (Fig. 6e). In comparison with various control groups, loss of GDF11 in the EN upregulated the mRNA of p21 to about fourfolds (Fig. 6g) and that of p53, another pro-senescence factor which is upstream to p21, to about twofold (Fig. 6h). Consistently, 2 target molecules of p53 were also upregulated by loss of GDF11 in the EN (Fig. 6c, d): tumor protein p53-induced nuclear protein 1 (Trp53inp1) which mediates cell cycle arrest or autophagy^41^ and its upregulation leads to the development of age-related cataracts^42^, and Pleckstrin homology-like domain family A, member 3 (Phlda3) which competitively suppresses Akt and indirectly regulates p53^43^. We also compared the in vitro transcriptome of the cellular senescence-related genes by bulk RNA-seq between GDF11^KO^ versus WT Neuro-2a cells_._ These genes were selected from GO database^40^ and their functions on cellular senescence were validated according to published studies. Our GO analysis revealed that among the top 20 senescence or ageing-related biological processes, they are mainly associated with cell adhesion, cell proliferation, response to cytokine stimulus and learning or memory (Supplementary Fig. 4g). Among 16 genes, 6 genes were downregulated and 10 upregulated by in vitro GDF11 deletion. Out of 10 upregulated genes, one is Nuak1, which controls cellular senescence shown previously^44^. These results suggest that loss of GDF11 affects a spectrum of senescence-associated biological processes and genes. In vitro loss of GDF11 also upregulated transcription of p21 (Fig. 6i), consistent with our in vivo data showing that loss of GDF11 in the EN upregulates p21 (Fig. 6c–g) and p53 (Fig. 6h). Collectively, GDF11 deletion both in mouse cortical EN in vivo and in Neuro-2a cells in vitro upregulates p21.

### In vivo deletion of GDF11 in excitatory neurons induced their own senescence requires p21

**Fig. 7:**
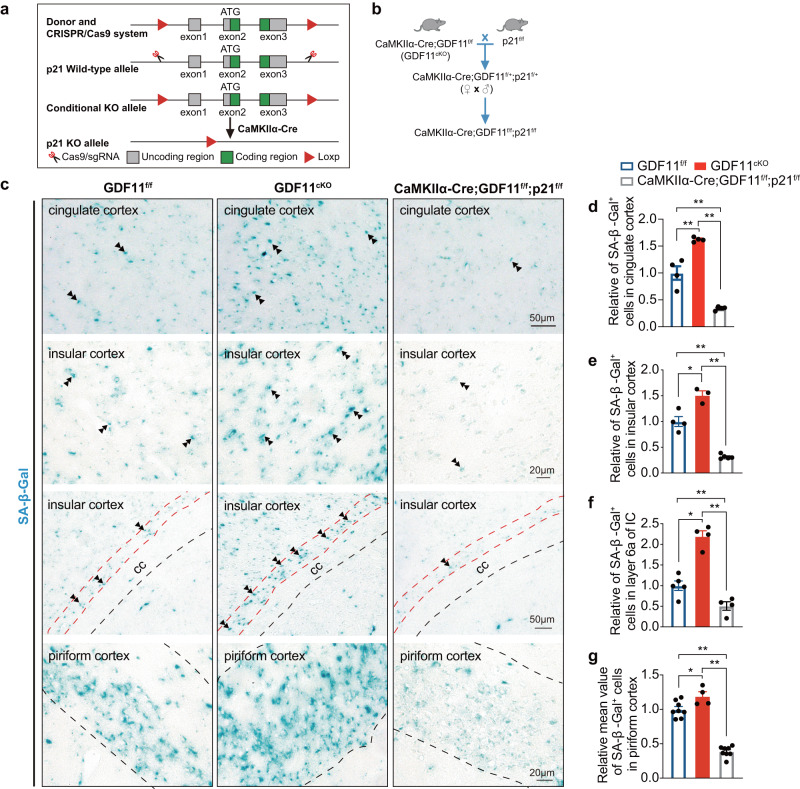
In vivo selective deletion of GDF11 in excitatory neurons induced their own senescence requires p21. **a**, **b** Genetic strategy for generation of p21^f/f^ mice (**a**) and CaMKIIα-Cre; GDF11^f/f^; p21^f/f^ mice (**b**) to selectively delete both GDF11 and p21 in CaMKIIα^+^ neurons through Cre/Loxp system. **c**–**g** Representative images (**c**) and quantification (**d**–**g**) of the SA-β-Gal^+^ cells in the cingulate cortex (**c**, up, and **d**, *n* = 4 per group), layers 4 and 5 (**c**, middle, and **e** GDF11^f/f^, *n* = 4; GDF11^cKO^, *n* = 3; CaMKIIα-Cre; GDF11^f/f^;p21^f/f^, *n* = 5), layer 6a (**c** middle, and **f** layer 6a is the deep layer cortex near the corpus callosum (CC), GDF11^f/f^, *n* = 5; GDF11^cKO^, *n* = 4; CaMKIIα-Cre; GDF11^f/f^;p21^f/f^, *n* = 4) of the insular cortex (IC), and layers 2 and 3 of the piriform cortex (**c** down, and **g** the dashed lines indicate the borders of layers 2 and 3, GDF11^f/f^, *n* = 8; GDF11^cKO^, *n* = 4; CaMKIIα-Cre; GDF11^f/f^;p21^f/f^, *n* = 8) of CaMKIIα-Cre; GDF11^f/f^;p21^f/f^ or GDF11^cKO^ or GDF11^f/f^ mice aged 17 M. Examples of the SA-β-Gal^+^ cells are indicated by double arrows. Scale bars, as shown on the images, 50 μm (**c**, up and middle) and 20 μm (**c**, middle and down). Data are presented as mean ± SEM. *P < 0.05, **P < 0.01. **d** (*F* (2, 9) = 72.52, *P* < 0.0001; GDF11^f/f^ versus GDF11^cKO^, *P* = 0.0006; GDF11^cKO^ versus CaMKIIα-Cre;GDF11^f/f^;p21^f/f^, *P* < 0.0001; GDF11^f/f^ versus CaMKIIα-Cre;GDF11^f/f^;p21^f/f^, *P* = 0.0004), **e** (*F* (2, 9) = 78.16, *P* < 0.0001; GDF11^f/f^ versus GDF11^cKO^, *P* = 0.0020; GDF11^cKO^ versus CaMKIIα-Cre;GDF11^f/f^;p21^f/f^, *P* < 0.0001; GDF11^f/f^ versus CaMKIIα-Cre;GDF11^f/f^;p21^f/f^, *P* < 0.0001), **f** (*F* (2, 10) = 49.87, *P* < 0.0001; GDF11^f/f^ versus GDF11^cKO^, *P* < 0.0001; GDF11^cKO^ versus CaMKIIα-Cre;GDF11^f/f^;p21^f/f^, *P* < 0.0001; GDF11^f/f^ versus CaMKIIα-Cre;GDF11^f/f^;p21^f/f^, *P* = 0.0347) and **g** (*F* (2, 17) = 102.8, *P* < 0.0001; GDF11^f/f^ versus GDF11^cKO^, *P* = 0.0227; GDF11^cKO^ versus CaMKIIα-Cre;GDF11^f/f;^p21^f/f^, *P* < 0.0001; GDF11^f/f^ versus CaMKIIα-Cre;GDF11^f/f^;p21^f/f^, *P* = 0.0001), One-way ANOVA with post Tukey multiple comparisons test. Source data are provided with this paper.

Having shown that in vivo selective deletion of GDF11 in the EN of CNS accelerates EN senescence and brain ageing preferentially in three brain regions and impairs object recognition memory and social cognition (Figs. 2 and 4), and these phenotypes are associated with upregulation of p21 (Figs. 5 and 6). Since it has been reported that neuronal senescence can be rescued by p21 deficiency^5^, we wonder whether loss of GDF11 in the EN-induced EN senescence also requires p21. To answer this question, we generated a GDF11 and p21 double knockout mouse line (CaMKIIα-Cre; GDF11^f/f^; p21^f/f^ mouse, Fig. 7a, b). SA-β-Gal staining and quantification showed that at age of 17 M, when compared with GDF11^f/f^ mice, loss of GDF11 alone in the EN (GDF11^cKO^ mouse) promoted EN senescence preferentially in the cingulate, insular and piriform cortices, similar to what described in Fig. 2. Importantly, loss of GDF11 alone in the EN accelerated EN senescence was rescued when p21 was deleted together with GDF11 (CaMKIIα-Cre; GDF11^f/f^; p21^f/f^ mouse, Fig. 7c–g), indicating that loss of GDF11 induced EN senescence requires p21. It is worthwhile to note that loss of p21 alone reduced cellular senescence to a level significantly less than GDF11^f/f^ mice at the same age, consistent with an essential role for p21 in neuronal senescence during chronological ageing. These results indicate that loss of GDF11 in the EN-induced EN senescence requires p21, suggesting a crucial role of p21 during EN senescence.

### Loss of GDF11 upregulates p21 via Smad2

**Fig. 8:**
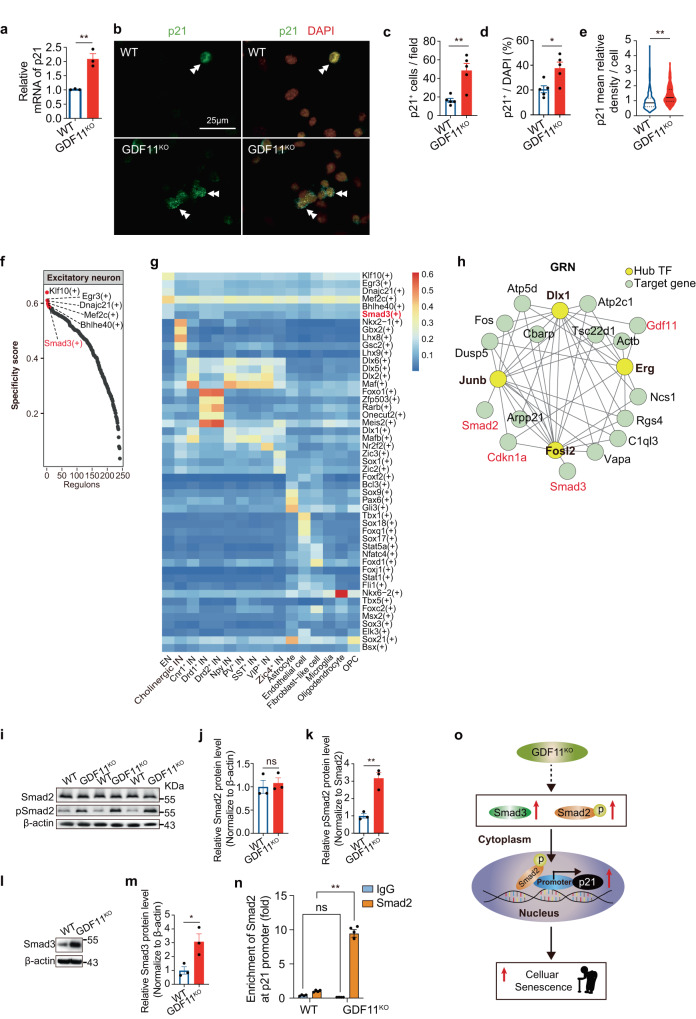
Loss of GDF11 upregulates pSmad2 and Smad3 and enhances Smad2 binding to the promoter of p21. **a** Quantification by qPCR of the relative p21 mRNA in the GDF11^KO^ and WT Neuro-2a cells (*n* = 3 clones). **b**–**e** Immunofluorescence representative images (**b**) and quantification of the number of p21^+^ cells per field (**c**, *n* = 5 fields/group), the proportion of p21^+^ cells (**d**, *n* = 6 fields/group) or the average gray value of p21 per cell (**e**, GDF11^KO^, *n* = 420 cells; WT, *n* = 280 cells) in the GDF11^KO^ and WT Neuro-2a cells. Scale bar, 25 μm. Examples of the p21^+^ cells are indicated by double arrowheads. **f**–**h** The same snRNA-seq data were used, as described in Fig. 5a. **f** Rank for regulons in the EN based on regulon specificity score (RSS). The EN were obtained from the Cg2 of the “KO” mice and the “Ctrl” mice aged 4–5 M. **g** Regulons activity analysis based on area under the curve (AUC) in the identified cell types in snRNA-seq of the “KO” mice and the “Ctrl” mice aged 4–5 M. The activity of regulon Smad3 (highlighted in red) is high in the EN. **h** Cytoscape network visualization of genes including GDF11, Cdkn1a (p21), Smad2, Smad3 (highlighted in red) and their transcription factors (TFs, yellow). **i**–**m** Representative images (**i** and **l**) and quantification by densitometry of western blot analysis of Smad2 (**j**), phosphorylated Smad2 (pSmad2, **k**) and Smad3 (**m**) in the total protein extracted from the GDF11^KO^ and WT Neuro-2a cells (*n* = 3 biological repeats/group). **n** ChIP-qPCR assessment of the enrichment of Smad2 at the promoter of Cdkn1a/p21 in the GDF11^KO^ and WT Neuro-2a cells (*n* = 3 biological repeats/group). **o** A proposed working model for loss of GDF11 on cellular senescence. Loss of GDF11 upregulates pSmad2, enhances nuclear entry of Smad2/3 tricomplex and then Smad2 binds to the promoter of p21 and promotes the pro-senescence factor p21 transcription, and eventually causes cellular senescence. Data are presented as mean ± SEM. *P < 0.05, **P < 0.01 and “ns” indicates not significant. **a** (*P* = 0.0037), **c** (*P* = 0.0033), **d** (*P* = 0.0157), **e** (*P* < 0.0001), **j** (*P* = 0.6648), **k** (*P* = 0.0040) and **m** (*P* = 0.0299), unpaired two-tailed *t* test. **n** (IgG: WT versus GDF11^KO^, *P* = 0.57; Smad2: WT versus GDF11^KO^, *P* < 0.001), two-way ANOVA with Sidak’s test. Source data are provided with this paper.

Since both our in vitro and in vivo data showed that loss of GDF11 enhanced transcription of p21, we then verified by qPCR that the transcription of p21 increased about twofold by in vitro GDF11 deletion (Fig. 8a). Immunofluorescence (Fig. 8b) further showed that the density (Fig. 8c), the proportion (Fig. 8d) of p21^+^ cells and single cell p21 immunofluorescence intensity (Fig. 8e) were all increased by in vitro GDF11 deletion, supporting that cellular senescence caused by loss of GDF11 is associated with p21 upregulation.

Having shown that loss of GDF11 in the EN of the CNS accelerates EN senescence, to explore its underlying molecular mechanism, our snRNA-seq analysis of 24803 single nuclei obtained from the Cg2 of 3 fGDF11^cKO^ and 3 GDF11^f/f^ mice revealed that in the EN, Smad3 was among the top regulons (Fig. 8f), and the regulon activity of Smad3 was enhanced in the EN (Fig. 8g). The gene and the transcription factor network analysis displayed a strong interaction network among GDF11, Smad2, Smad3 and Cdkn1a/p21 (Fig. 8h).

To carefully dissect the sequentially regulatory mechanism underlying the p21 upregulation caused by the loss of GDF11, we showed with western blot that in comparison with WT Neuro-2a cells, GDF11 deletion did not affect Smad2 (Fig. 8i, j) but increased both phosphorylated Smad2 (pSmad2) (Fig. 8i, k) and Smad3 (Fig. 8l, m). Since pSmad2 is necessary for nuclear entry of Smad tricomplex^45,46^ of which Smad3 is also a member^47^, we wonder whether Smad2 directly binds to the promoter of p21. By chromatin-immunoprecipitation q-PCR (ChIP-qPCR) technique we found that at p21 promoter Smad2 occupancy was dramatically enriched indeed (Fig. 8n), suggesting that loss of GDF11 enhances Smad2 binding to the promoter of p21. Collectively, we propose that GDF11 deletion upregulates Smad2 phosphorylation and facilitates nuclear entry of Smad2/3 tricomplex, then Smad2 can bind to the promoter of p21 and promotes transcription of p21, a pro-senescence factor for neuronal senescence (Fig. 8o).

## Discussion

Emerging evidence shows that the EN is the major cell type contributing to brain ageing and organismal ageing^7,11^, it remains unknown how the EN itself in the brain acquires senescence^8,21^. To explore this, using a specific antibody for GDF11, a putative rejuvenation factor^12–14^, we showed that GDF11 is predominantly expressed in the CaMKIIα^+^ EN in the adult mouse, marmoset and human brain. Since CaMKIIα is exclusively expressed in the post-mitotic EN throughout the adult CNS^48^, our results revealed that GDF11 is predominantly expressed in the post-mitotic EN in the adult brain in an evolutionarily conserved manner.

GDF11 exhibits protective roles in a variety of tissues including anti-ageing on skin^49^, against vascular calcification^50^ and spinal cord injury^51^. To explore whether GDF11 plays a role in excitatory neuronal senescence and brain ageing, we adopted genetic deletion strategies. Considering GDF11^‒/‒^ mouse, in which GDF11 was systemically deleted, is embryonic lethal^18^, to conditionally delete GDF11, we generated a new GDF11^f/f^ mouse line. In GDF11^cKO^ (GDF11^f/f^; CaMKIIα-Cre) mouse, Cre/Loxp recombination occurs since the third postnatal week^19^ thus only GDF11 in the post-mitotic EN was exclusively deleted with minimal affecting CNS development. We found that specific deletion of GDF11 in the post-mitotic EN of the mouse CNS caused an increase in SA-β-Gal^+^ senescent cells, a surrogate biomarker of senescent cells^3,20^, preferentially in the insular, piriform and cingulate cortices. This result is consistent with a large-population-based human cohort study that the insular cortex is among the very few cerebral cortical regions whose gray matter volume declines with progressive ageing and exhibits positive association with physical and general health^52^. Furthermore, we confirmed that the SA-β-Gal^+^ senescent cells were predominantly the post-mitotic CaMKIIα^+^ EN. Taking into accounts of the possibly wide expression of the versatile receptors of GDF11, GDF11 deletion-induced EN senescence takes place likely in manners of autocrine and/or paracrine. In addition, we showed that in vivo selective deletion of GDF11 in the EN pruned and shortened the apical dendrites and reduced the mushroom spines of the EN in the young mice, manifesting the features of the senescent cortical EN (pyramidal neuron) of the aged human^53,54^. In parallel, our in vitro results showed that loss of GDF11 impeded mitochondria, enlarged cell nuclear size, might impair lipid metabolism and accumulated both lipofuscin and neurosecretory granules in their cytoplasm-all these have been established previously as typical metabolic and ultrastructural features of cellular senescence^3^. Selective deletion of GDF11 in GDF11^KO^ Neuro-2a cells in vitro (bulk RNA-seq) and in the fGDF11^cKO^ prefrontal cortical EN in vivo (snRNA-seq) drives transcriptional programs which consistently point to neuronal senescence, manifesting the features of senescent neurons in the naturally aged mouse^10^ and human^6,11^ brain. Our RNA-seq data also suggest that GDF11 exhibits pro-survival effects to cells, consistent with previous reports that GDF11 improves the survival of both neuronal and oligodendroglial cells in the brain^51^ and islet β-cells in the pancreas^55^, and GDF11 also maintains telomere length in Neuro-2a cell line^56^, suggesting that GDF11 exhibits pro-survival effects in both proliferative cells and post-mitotic neurons and involves cellular senescence. Collectively, through systematic, multi-parametric approaches of in vitro and in vivo, our results have established a causality between the loss of GDF11 and the EN senescence and have revealed the first molecular mechanism underlying the EN senescence that loss of GDF11 in the EN accelerates EN senescence preferentially in the insular, piriform and cingulate cortices. These results indicate that the endogenous GDF11 in the post-mitotic EN is required for them to resist neuronal senescence.

It remains unclear whether neuronal senescence leads to cognitive decline^8^. Having shown that loss of GDF11 in the EN causes EN senescence, we further ask whether induction of EN senescence is sufficient to affect their function. Indeed, in response to targeted deletion of GDF11 in the post-mitotic EN of cingulate gyrus in the prefrontal cortex, one of the brain regions in which accelerated EN senescence was induced by loss of GDF11 in the EN shown in this work and which is preferentially associated with age-dependent cognitive impairment shown previously^4^, our whole-cell patch clamp recording showed that the cortical EN with loss of GDF11 in the young mouse brain exhibited hyperexcitability and imbalance between excitation and inhibition, manifesting a functional signature of the aged cortical neurons^7,11^. To further explore whether induction of EN senescence can translate into cognitive deficits, we tested a battery of behavior assays and found that in mice, either focal or systemic deletion of GDF11 exclusively in the EN impaired object recognition memory and social cognition, but did not affect their locomotion, anxiety level and despair behavior. These results indicate that induction of EN senescence by loss of GDF11 is sufficient to cause cognitive decline, providing the first loss of function evidence which links induced neuronal senescence with impeded cognition. Our work is consistent with a recent gain of function study which shows supplementing GDF11 improves memory in the aged mice^57^.

**Fig. 9:**
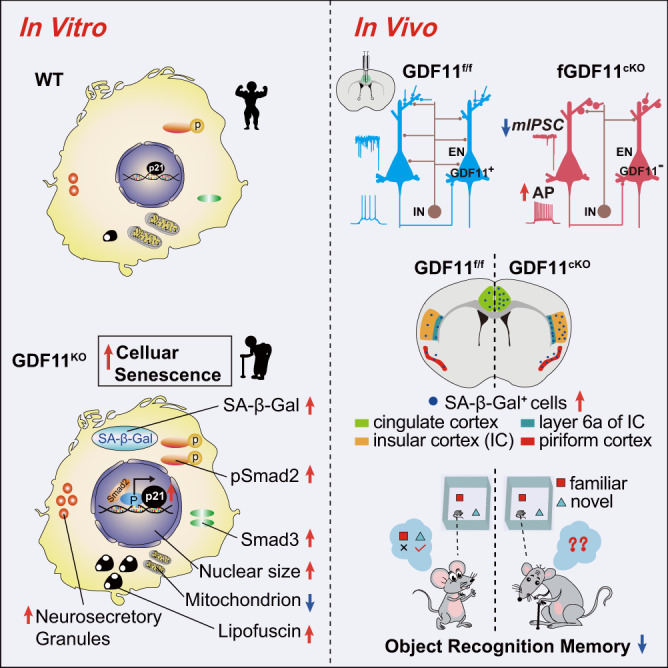
Schematic summary of the GDF11 effects on excitatory neuronal senescence and brain ageing. Evidence of both in vitro (left) and in vivo (right) indicates that growth differentiation factor 11-Smad2/3-p21 pathway acts as a brake on excitatory neuronal senescence and brain ageing.

Having established a causality between the loss of GDF11 and the EN senescence, to further explore its underlying molecular mechanisms, we compared the transcriptomes of cells in which GDF11 was deleted both in vitro and in vivo with the control, and found that loss of GDF11 both in Neuro-2a cells in vitro and in the mouse cortical EN in vivo upregulated pro-senescence factor p21, a two-faced genome guardian which can mediate cellular senescence in a p53-dependent manner or mediate genomic instability or even carcinogenesis in a p53-deficient environment^58^. The abundance of both p53 and p21 in the EN, as shown in our results, and their post-mitotic feature, render the EN less likely to cause carcinogenesis by loss of GDF11. Loss of GDF11 upregulated p21 at both mRNA and protein level. In vivo selective deletion of GDF11 in the cerebral cortical EN also upregulated transcription of p53, another pro-senescence factor^46^ which is upstream to p21, and 2 target molecules of p53, Trp53inp1 and Phlda3. These results support that loss of GDF11-induced EN senescence is associated with upregulation of p53 and p21, consistent with the established signaling pathway^58,59^. Interestingly, we found that loss of GDF11 also upregulated pSmad2, which was reported to promote the assembly and nuclear import of the Smad tricomplex and alter the transcriptional activity of target genes^45–47^. Moreover, our ChIP-qPCR data revealed that the Smad2 occupancy was enriched at the promoter of p21 in GDF11^KO^ cells. It has been shown previously that p21 causes cellular senescence in a dose-dependent induction^60^ and mediates cell cycle arrest upon DNA damage and prevents cell cycle progression^61^. Furthermore, p21 upregulation mediates senescence of both post-mitotic cortical neurons^5^ and post-mitotic dopaminergic neurons^40^. We further show here that loss of GDF11 in the EN-induced EN senescence requires p21, highlighting a crucial role of p21 during the EN senescence. Based on our results and previous reports, we propose here that loss of GDF11 upregulates pSmad2 and likely facilitate nuclear entry of Smad tricomplex, and then Smad2 binds to the promoter of p21 and enhances p21 transcription, and eventually causes senescence of the post-mitotic EN (Fig. 9).

Our results suggest that loss of GDF11 in the EN accelerated EN senescence and brain ageing might involve multiple mechanisms. Loss of GDF11 in the EN shortening lifespan is compelling, considering the EN only occupy a small proportion of cells in the brain, further studies in future will unveil new insightful mechanisms underlying EN senescence and brain ageing.

This work identifies GDF11-Smad2/3-p21 pathway as a brake on EN senescence and brain ageing and reveals the first molecular mechanism underlying the EN senescence. GDF11 deletion in the post-mitotic EN accelerates senescence of the post-mitotic EN preferentially in the insular, piriform and cingulate cortices and induces their own hyperexcitability. Moreover, GDF11 deletion-induced EN senescence, which requires p21, is sufficient to accelerate brain ageing, cause cognitive decline and shorten lifespan. Mechanistically, loss of GDF11 accelerates EN senescence by activating Smad2/3-p21 pathway. This study reveals a potential target to slow brain ageing by manipulating GDF11 in the EN.

## Methods

### Experimental model and subject details

#### Mice

Male ICR mice (Laboratory Animal Center of Zhejiang Academy of Medical Sciences) at age of 3 months (M), 9 M and 36 M, male C57BL/B6 wild‐type (WT) mice (Shanghai Slac Laboratory) at age of 3 M and 10 M, male GDF11-flox mice (GDF11^f/f^, mice carrying the “floxed” GDF11 gene) at age of 3 M, 10 M and 17 M, male GDF11^f/f^ x CaMKIIα-Cre mice (GDF11^cKO^) at age of 10 M and 17 M, male CaMKIIα-Cre; GDF11^f/f^; p21^f/f^ mice at age of 17 M, and male mice, at age of 4–5 M, of AAV-mediated focal GDF11^cKO^ in the cingulate gyrus were used. For survival curve study, male mice were bred and maintained in conditions same to the above-mentioned mice until they died naturally. Mice were housed in an environment of suitable temperature (25 °C) and humidity (typically 50%) under a 12 h light–dark cycle (light on from 7 am to 7 pm) with accessing to food and water ad libitum. All experimental protocols were approved by the Animal Care and Use Committee of the animal facility at Zhejiang University. The studies with mice were approved by Medical Ethics Review Committee of Zhejiang University School of Medicine (ETHICS number: 14660).

#### Marmoset brain tissue

The brains of two female common Marmosets (Callithrix jacchus, 260 g and aged 62 M; and 245 g and aged 70 M) were provided by Li-Xia Gao Lab and were post-fixed in 4% paraformaldehyde (PFA) for about 12 h after they were transcardially perfused. The studies with common Marmosets were approved by Medical Ethics Review Committee of Zhejiang University School of Medicine (ETHICS number: ZJU20210053).

#### Human brain tissue

Human brain tissues were obtained through a research protocol approved by the Zhejiang University Institutional Review Board. The informed consent was obtained for all the human samples in this study. Para-epileptic cortical tissues were obtained from two patients, aged 23 (female) and 24 (male) years, respectively, who were diagnosed with intractable epilepsy and the focus of epilepsy had to be removed surgically. In addition, the frontal lobe tissues were obtained from four patients, aged 23 (male), 52 (male), 54 (female) and 60 (male) years, respectively, who suffered acute brain injury and the peri-lesion cortical tissues were used. In this study, the human brain tissues were fixed in 4% PFA for 4 h and the sections were cut followed by immunofluorescence staining procedure. The studies with human samples were approved by Medical Ethics Review Committee of Zhejiang University School of Medicine (ETHICS number: IIT20220453B).

### Cell culture

Neuro-2a (ATCC) or HEK 293 T (ATCC) cells were cultured in high‐glucose DMEM (Gibco) supplemented with 50 U/ml penicillin-streptomycin (P/S) and 10% fetal bovine serum (FBS, Gibco). The maintaining condition for all cell cultures was at 37 °C with 5% CO_2_. To express GDF11 or GDF8 protein, the plasmids (pMs-GDF11-Flag-Myc, phGDF11-Flag, pMs-GDF11-eGFP-Flag or pMs-GDF8-Flag-Myc, phGDF8-Flag) were transfected into Neuro-2a or HEK 293 T cells using transfectamine 2000 (Thermo Fisher Scientific) according to the manufactured protocol with appropriate cell confluence (> 50%) after plating. After transfection, the cells were cultured for 48 h and harvested for RT-PCR, western blot analysis, immunofluorescence labelling or senescence-associated β-galactosidase (SA-β-Gal) staining. For in vitro experiments, the individual data points were obtained from a combination of three independent biological repeats.

### Generation of GDF11f/f, GDF11cKO, p21f/f and CaMKIIα-Cre, GDF11f/f;p21f/f mice

#### GDF11^f/f^ mice

Flox sites of GDF11^f/f^ mice were designed following this strategy: 5’-arm-loxp1-exon 2-loxp2-3’-arm. The flox sites were inserted on both sides of exon 2 of GDF11 gene using the CRISPR/Cas9 system. A single cut was performed to target exon 2 of GDF11 and GDF11-specific gRNA sequence of mice was designed using sgRNA designer website (https://chopchop.cbu.uib.no/) as indicated below: 5’-ggggtgaaggtatcagttag-3’. Loxp1 insert position and sequence (in brackets) was: CTAGGAGAAATGTTATAGGGGATAGTATAGATCAG(cgttcggggaattcATAACTTCGTATAATGTATGCTATACGAAGTTATgtcggtgc)AAGGCCTAACAGACTCATCATCCTAGTGA. Loxp2 insert position and sequence (in brackets) was: CTGGATTTGGGGTGAAGGTATCAGTTA(cactcgctATAACTTCGTATAATGTATGCTATACGAAGTTATactagtcgttc)GGGAGATCCGTGGGAACACAAAG.

The validation primers designed according to the loxp site were below:

GDF11-wt-F: 5’-GGGTGAAGGTATCAGTTAGTGGG-3’;

GDF11-wt-R: 5’-GCTCTCATGTCGCTCAGGTTG-3’;

GDF11-flox-F: 5’-TGAAGGTATCAGTTACACTCGCTAT-3’;

GDF11-flox-R: 5’-TGAGCACTTCTCTTGACATCTAGAC-3’.

The PCR production of “WT primer pairs” was 167 bp and “flox primer pairs” was 349 bp. These primers were used to distinguish WT ( + / + , 167/167 bp), heterozygous (f/+, 349/167 bp) and homozygous (f/f, 349/349 bp) GDF11^f/f^ mice.

GDF11^cKO^ mice were generated using the Cre/Loxp system. GDF11^f/f^ mice were crossed with CaMKIIα-Cre mice using a three-step backcrossing procedure to obtain GDF11^f/f^; CaMKIIα-Cre (GDF11^cKO^). The validation primers with forward (CaMKIIα-Cre-F) primer (5’- TGCCCAAGAAGAAGAGGAA-3’) and reverse (CaMKIIα-Cre-R) primer R (5’- TTGCAGGTACAGGAGGTAGTC-3’) were designed to distinguish whether the target sequence contains the sequence of cyclization recombination enzyme (Cre) or not. To test the knockout efficiency of GDF11^cKO^, genomic DNA was extracted from the cortical tissue of WT, GDF11^f/f^ and GDF11^cKO^ mice using a TIANamp Genomic DNA Kit (TIANGEN). Primers were designed according to the target sites with forward (F) primer (5’-CAGGTGCAGCAAAGAGCATT-3’) and reverse (R) primer R (5’-GCTCTCATGTCGCTCAGGTT-3’). PCR was performed to determine the knockout efficiency by analyzing the different size bands on 1.2% agarose gel. PCR product should be 1091 bp in WT mice and 1197 bp in GDF11^f/f^ mice, whereas it should be 312 bp if exon 2 of GDF11 gene was deleted in GDF11^cKO^ mice.

#### p21^f/f^ mice

Flox sites of p21^f/f^ mice were designed following this strategy: 5-arm-loxp1-exon 1- exon 2- exon 3-loxp2-3-arm. The flox was flanked on the exon 1 to exon 3 of p21 gene using the CRISPR/Cas9 system. p21-specific gRNA sequences of mice were designed using sgRNA designer website (https://chopchop.cbu.uib.no/) as indicated below: gRNA1, 5’-GTTTGGGCCTCTCCGAGAGG-3’, PAM, TGG, and gRNA2, 5’-TCCCTGGGAGTATTGAACCT-3’, PAM, GGG. Loxp1 insert position and sequence (in brackets) was: CCACCGCATCGCATTGTCTGAGTA GGTGCT TAAGGG ATCCAG ATCTGG GCCC(ATAACTTC GTATAGCATACATTATACGAAGTTAT)ATGTTCTTGCCCAAGGTCAGTTGGTCTCGGAGAG. Loxp2 insert position and sequence (in brackets) was: CTAGCCAGTACACCCCCTAAAAACCATGGCCAACAAACTGAAGTCCCTCTGAGGCGGAAAGAACCAG(ATAACTTCGTATAGCATACATTATACGAAGTTAT)GGGCCGAATTCGATATCCTGCAGGGACTAACAGAAGAACCCGTTGTGAGGTTCAATACTCCCAGGGAGTGTCAGAGTCT. The validation primers of p21^f/f^ mouse designed according to the loxp site was below:

p21-floxp-F: 5’-CAGGACTTCAGACTGCGCTC-3’;

p21-floxp-R: 5’-TGTGGCATGCAGGTGGATTG-3’.

The PCR production of “p21-floxp primer pair” was 186 bp (WT) or 291 bp (floxp). The primers were used to distinguish WT ( + / + , 186/186 bp), heterozygous (f/+, 291/186 bp) and homozygous (f/f, 291/291 bp) p21^f/f^ mice.

CaMKIIα-Cre, GDF11^f/f^; p21^f/f^ mice were generated through Cre/Loxp system. p21^f/f^ mice were crossed with GDF11^cKO^ mice to obtain CaMKIIα-Cre, GDF11^f/f^; p21^f/f^ mice. The forward (CaMKIIα-Cre-F) primer (5’-TGCCCAAGAAGAAGAGGAA-3’) and reverse (CaMKIIα-Cre-R) primer R (5’-TTGCAGGTACAGGAGGTAGTC-3’) were designed to distinguish whether the mice contained the sequence of Cre enzyme or not.

### Focal AAV injection into the cingulate gyrus of GDF11f/f mice

AAV-CaMKIIα-Cre-EGFP (“KO virus”, AAV9, 3.55 × 10^14^ vg/ml) and AAV-CaMKIIα-EGFP (“Ctrl virus”, AAV9, 6.90 × 10^13^ vg/ml) were made by Shandong Vigene Biosciences Co. Ltd. (Shandong, China). After anaesthetized using 1.5% pentobarbital sodium, mice were stereotaxic injected with a volume of 0.15–0.2 μl virus solution (according to viral titer and expression strength). The injected speed of virus was 0.02 μl/min at each location, and the syringe was not removed until 5 min after the infusion to allow virus diffused sufficiently. A heating pad was used to maintain core body temperature at about 36°C. The viral injection sites were bilateral cingulate gyrus (AP, +0.7 mm; ML, ±0.3 mm; DV, –1.2 mm).

### Behavioral assays

All behavior tests were performed between 9 am and 6 pm without the experienced investigators being present in the testing room treated to avoid noise interference. All behavioral assays were carried out on male mice. Experienced investigators analyzing the data of behavior were blind to the mice grouping. The following behavioral tests were carried out in the order as described below.

#### Open filed test (OFT)

OFT was used to assess locomotion. It was conducted in a white open field box (50 × 50 × 50 cm) with opaque polyvinyl plastic. The field was subdivided into 16 (4 × 4) identical sectors, and the 4 central squares were defined as central sector and the remained surrounding squares were defined as peripheral sector. Mice were introduced into the center of the open field and allowed to freely explore for 10 min as a standard time. Occasionally the exploration time was extended to 30 min and only the data obtained from the first 10 min was used. Open field box was thoroughly cleaned with ethanol (70%) before experimental use and between trials. A video tracking system (ANY-Maze software) was used to record the time spent in and entries into (anxiolytic indicator) the central sector.

#### Passive avoidance test (PAT, light–dark transition test)

To evaluate aversive stimuli-driven memory and learning, PAT was conducted. The light–dark box (21 × 42 × 25 cm) was divided into a large light field (two thirds, 300 lux) and a small dark field (one third, 5 lux). Both sectors were connected through a small doorway (5 × 3 cm). Mice were introduced into the dark field and let freely explored in the two fields for 10 min. Time spent in the light field was recorded.

#### Elevated plus maze (EPM)

EPM was performed to assess anxiety-like behavior. The cross-shaped EPM consists of two open and two closed (each 30 × 5 cm) arms with walls (15 cm height) and a crossing central zone (5 × 5 cm). The EPM elevated to a height of 50 cm from the floor. Mice were placed in the crossing central zone facing an open arm and allowed to freely explore in the EPM for 5 min. Any-Maze behavioral tracking system was used to record the time spent in and the number of entries into the open or closed arms. The EPM apparatus was cleaned thoroughly using ethanol (70%) between trials.

#### Novel object recognition test (NORT)

NORT was conducted to test object recognition memory in a room adjacent to the animal house and was performed in a white open field box (50 × 50 × 50 cm) with opaque polyvinyl plastic. Animal’s behaviors were recorded and scored off line by using Any-Maze software. During the habituation session, mice were introduced to the open field box for 5 min without any objects and allowed to explore freely. During the training session, mice were allowed to explore for 5 min with two identical objects in the open field box. The objects used in this experiment were the same metal blocks called ‘familiar’ objects. The time that mice spent on exploring each familiar object was recorded and this procedure was defined as the training session. The memory test was conducted 24 h after the training session. During the testing session, mice were left to explore both objects for 5 min, in which one of the familiar objects was replaced with a novel object different in its color, shape, and texture and was put in the same place. The time spent on exploring the novel (TN) and the familiar (TF) objects were recorded. Mice were regarded to be exploring when they were contacting with the object (sniffing, biting) or facing the object (distance <2 cm). The box and objects were cleaned with ethanol (70%) before experimental use and between tests. The object recognition memory was evaluated by comparing the percentage of the exploration time spending on the novel (% of exploration time = TN/[TN + TF] x 100). The object recognition memory performance was also assessed by the novel object discrimination index (NODI = [TN-TF]/[TN + TF]).

#### Three-chamber test (3CT)

To evaluate sociability and social cognition, 3CT was performed in a three-chambered box (62 cm length × 42 cm width × 22 cm height) consisted of the opaque polyvinyl plastic. During the habituation phase, mouse was placed in the middle chamber and allowed to explore three chambers freely for 10 min with one empty vertical metal wire cage in the corner of each extreme chamber. The first session aims to test the sociability, and a stranger (S1) mouse was placed into one of the vertical metal wire cages and the other cage was empty object (O). Then the mouse was placed in the middle chamber and allowed to explore freely all chambers for 10 min. The second session is to test its social cognition, and a new stranger mouse (S2) was placed in the chamber and on the other end was S1. In this session, the mouse was allowed to explore freely for 10 min. S1 and S2 at same age and with same sex to the mice to be tested were used. The time spent in each chamber was recorded and analyzed using Any-Maze software. The sociability was evaluated by the time preference index (S1-O) and the social cognition was calculated by the time preference index (S2-S1).

#### Tail suspension test (TST)

TST was used to test despair behavior. The TST apparatus is consisted of white box with an open front. The mouse was suspended using adhesive tape placed less than 1 cm from the tip of the tail and 50 cm above the surface. The video was used to record the mouse behaviors for 6 min. The immobility time was assessed during the last 5 min.

### Senescence-associated β-galactosidase staining

Senescence-associated β-galactosidase (SA-β-Gal) Staining Kit (Beyotime) was used to stain senescent cells. To avoid the possible variation caused by staining procedure, brain sections for comparison (e.g., brain sections of GDF11^cKO^ and GDF11^f/f^ mice) were mounted on the same slide and then subjected to SA-β-Gal staining simultaneously. X-Gal was specifically catalyzed into a blue colored product at pH 6.0 by β-galactosidase which was expressed in senescent cells. After an incubation at 37 °C overnight in incubator without CO_2_, an Olympus BX53 microscope was used to image the blue colored product which was directly proportional to β-galactosidase expression. The number or mean grey value of blue colored positive cells was calculated. After SA-β-Gal staining, sections were washed four times (5 min) in 0.01 M PBS and further performed a standard immunofluorescence staining of NeuN (1:1000, Millipore) or immunohistochemistry staining of CaMKIIα (1:500, Abcam). Imaging of immunofluorescence and SA-β-Gal staining were performed sequentially under fluorescent or bright field respectively using an Olympus microscope (BX53).

### Brain slice preparation and electrophysiological recordings

Brain slice preparation was performed as previously described^62^. Mice were anesthetized with intraperitoneal (i.p.) injection of sodium pentobarbital (50 mg/Kg), and then were transcardially perfused with ice-cold sucrose-based ACSF (NaCl was replaced by sucrose) and decapitated rapidly. Coronal sections (300 μm) containing the cingulate gyrus (Cg) were obtained from GDF11^f/f^ mice aged 4 M, which received a focal bilateral injection into the Cg2 with “AAV-CaMKIIα-Cre-EGFP, KO virus” or “AAV-CaMKIIα-EGFP, Ctrl virus” at age of 2 M and survived further for 2 M. Brain slices containing the Cg were cut in this sucrose-based ACSF with a vibratome (VT−1200S, Leica), and then transferred immediately to an incubation chamber filled with normal ACSF (see below) and maintained at 34 °C. After 45-60 min incubation, slices were maintained at room temperature until use. An infrared-differential interference contrast (IR-DIC) microscope (BX-51WI, Olympus) was used for visualization of cortical cells. The normal ACSF contained (in mM): 126 NaCl, 2.5 KCl, 26 NaHCO_3_, 1.25 NaH_2_PO_4_, 2 MgSO_4_, 2 CaCl_2_, and 25 dextrose (315 mOsm, pH 7.4). All solutions for slice preparation and recording were bubbled with 95% O_2_ and 5% CO_2_.

Whole-cell recordings from the EN were performed using patch pipettes with impedance of 4–7 MΩ. The pipette solution contained (in mM) 140 K-gluconate, 3 KCl, 2 MgCl_2_, 10 HEPES, 0.2 EGTA, 2 Na_2_ATP and 0.2% biocytin (285–295 mOsm, pH 7.2). We employed a Multiclamp 700B amplifier (Molecular Devices) and a Micro3 1401 analog-to-digital converter together with Spike2 software (version 8, Cambridge Electronic Design) for data acquisition. Positive current pulses (50−100 pA per step, 500 ms in duration) and negative current pulses (−30 pA per step, 500 ms) were injected to examine the electrophysiological properties such as F-I curve and input resistance when the cell was at resting membrane potential (RMP). The liquid junction potential (~15 mV) was not corrected for the membrane potential shown in the text and figures.

Current recordings were obtained with a Cs^+^-based/high-Cl^-^ pipette solution containing (in mM) 120 CsCl, 10 TEA-Cl, 10 HEPES, 1 EGTA, 10 sodium phosphocreatine, 4 MgATP, 0.3 Na_3_GTP, 2 QX314-Cl, 0.4% biocytin (299 mOsm, pH 7.25). To obtain the miniature excitatory postsynaptic currents (mEPSCs), we added 50 μM picrotoxin (PTX) to block fast GABAergic synaptic transmission and 1 μM TTX to block sodium spikes. To obtain the miniature inhibitory postsynaptic currents (mIPSCs), we added 1.5 mM kynurenic acid (Kyn) to block ionotropic glutamate receptor together with 1 μM TTX. The recorded cells were held at −70 mV in voltage clamp mode. For PPR experiments, an extracellular glass electrode was placed ~100 μm away from the recorded neuron. The stimulus intensity was 2-3 times the threshold current. The inter-stimulus-interval was 50 ms.

Data analysis was performed using Spike2 and MATLAB (MATHWORKS). A successful AP was defined when the amplitude surpassed 30 mV. The AP threshold was defined as the membrane potential when the rising phase of the first AP (AP1) in an AP train (10–50 Hz) evoked by positive pulse injection reached 20 V/s. The rheobase current was defined as the threshold current when the current step evoked a single action potential. The sag ratio was obtained from the peak amplitude of the hyperpolarization (−90 pA) divided by the steady-state *V*_m_ change during the negative pulse injection. For group comparison, Shapiro-Wilk test was used for data normality test. Two-sample Student’s t test was used if they were normally distributed, otherwise Mann–Whitney test would be employed. Data were presented as mean ± SEM in the text and figures.

### Dendritic morphology image and analysis

Biocytin (0.2%) was added into the internal solution of the patch pipette, and post hoc streptavidin staining (Alexa Fluor 555-conjugated, Invitrogen S32355, 1:500) was performed to visualize the whole cell morphology. For 3D-reconstruction of the labeled cells, z stack images (0.75 μm per image) were acquired with an air objective (×20) on a confocal microscope (Nikon A1 plus, Japan) and processed using ImageJ. The soma diameter and distance to the pia were obtained from these reconstructed cells in ImageJ. The surface membrane area (soma and dendrites) of the pyramidal neurons (PNs) was obtained from streptavidin staining confocal images by using the 3D Objects Counter function in ImageJ. For spine count, z stack images (0.5 μm per image) were taken with a 100× oil-immersion objective on the confocal microscope and processed using NeuronStudio.

### Single nucleus suspension and library preparation

#### Collection of the mouse brain

GDF11^f/f^ mice aged 2 M received bilateral focal injection into the cingulate gyrus 2 (Cg2) of “AAV-CaMKIIα-Cre-EGFP, KO virus” or “AAV-CaMKIIα-EGFP, Ctrl virus”, and after 2 M AAV injection, mice aged 4 M were euthanized by neck dislocation. Mouse brain was dissected on an ice-cold board to obtain the bilateral Cg2 and transferred to cryogenic vials (Nest, 607001). Samples were snap-frozen and stored in liquid nitrogen until nuclear extraction was performed.

#### Single nucleus suspension preparation

Single nucleus (sn) suspension preparation was performed^63^. In brief, frozen brain tissue was thawed and homogenized by 25–50 strokes of the loose pestle (pestle A) using Dounce homogenizer (TIANDZ) in an ice box with 1 ml of buffer A (10 mg/ml BSA (Ambion), 250 mM sucrose (Ambion), 0.12 U/μl RNasin Plus (Promega, N2115), 5 mM MgCl_2_ (Ambion), and 1× Complete Protease Inhibitor Cocktail (Roche, 11697498001). The mixture was filtered using a 100 µm cell strainer and further homogenized by 25 strokes of the tight pestle (pestle B) with 750 μl buffer A containing 1% Igepal (Sigma, CA630). Then, the samples were filtered through a 40 µm strainer and centrifuged at 500× *g* for 5 min at 4 °C to pellet the nuclei. Nuclei were then resuspended with buffer B (10 mg/ml BSA, 320 mM sucrose, 2 mM magnesium acetate, 3 mM CaCl2, 10 mM Tris-HCl, 0.1 mM EDTA, 1 mM DTT, 0.12 U/μl RNasein and 1× Complete Protease Inhibitor Cocktail) and the concentration used for library preparation was 1000 nuclei per μl.

#### snRNA-seq library preparation

snRNA-seq libraries were prepared using the DNBelab C Series Single-Cell Library Prep Set^64^ (MGI, 1000021082). In brief, single-nucleus suspensions were converted to barcoded snRNA-seq libraries through the generation of droplet, breakage of emulsion, collection of bead, reverse transcription and cDNA amplification. Indexed libraries were performed based on the manufacturer’s protocol. The Qubit ssDNA Assay Kit (Thermo Fisher Scientific, Q10212) was used to measure the concentration. Then, the prepared libraries were sequenced using the DNBSEQ-T1 or DNBSEQ-T7 sequencer at the China National GeneBank (Shenzhen, China) and the sequencing strategy was below: 41-bp read as read 1 and 100-bp read as read 2.

### snRNA-seq data processing

#### Raw data processing

Raw sequencing reads from DIPSEQ-T1 were filtered and demultiplexed using PISA (v0.2) (https://github.com/shiquan/PISA). Reads were aligned to genome using STAR (v2.7.4a)^65^ and sorted by sambamba (v0.7.0)^66^. Cell versus gene unique molecule identifier (UMI) count matrices were generated by custom script. Count matrices were processed using the Seurat package (v4.0.6)^67^ in R (v4.0.5). Outlier cells were filtered out based on the distribution of the counts of RNA features and expression of mitochondrial genes. For our data, cells with less than 500 genes detected, greater than 5000 genes detected or more than 5% mitochondrial reads were excluded. Then, the filtered dataset was normalized and scaled and PCA was calculated. The first 20 PNs were used to construct a SNN network and a graph-based clustering approach, Louvain algorithm, was applied to identify cell clusters with the resolution set to 0.8. Finally, UMAP was applied to visualize the clustering result in 2D space.

#### Cluster marker analysis and cell type annotation

To annotate each cluster to a specific cell type, we selected some classic markers for immune cells and used violin plots and dot plots to annotate cell types. The following genes were used for cell type annotation: Slc1a3, Mfge8, Gfap, (Astrocytes); Flt1, Cldn5, Pecam1 (Endothelial cells); Dcn (Fibroblast-like cells); Hexb, C1qb, Tmem119 (Microglia); Cldn11, Mag, Mbp (Oligodendrocytes); Pdgfra, Cspg4 (Oligodendrocyte precursor cells); Slc17a7, Slc17a6 (excitatory neurons, EX), Adrb1 (L3 EX), Etv1, Bcl11b (L5 EX), Tle4, Htr2c (L6 EX); Slc6a1, Slc32a1, Gad1, Gad2 (inhibitory neurons, IN), Pvalb, Vip, Sst, Cnr1, Npy, Drd1, Drd2 (specific IN).

#### Differential expressed genes (DEGs) analysis and GO enrichment

We performed DEG analysis using the “FindMarkers” function in Seurat R package. The cell populations we compared were input as ident.1 and ident.2, respectively. The fold changes of the average expression level of genes between the selected cell populations were calculated. To find the function of upregulated or downregulated genes (adjusted *P* < 0.01, Fold change >2), we used the function enrichment GO (OrgDb = org. Mm. eg. db, ont = ‘BP’, *P* Adjust Method = ‘BH’, *q* value Cutoff = 0.2, *P* value Cutoff = 0.05) of clusterProfiler (version 3.0.4)^68^ R package.

#### Regulatory network inference and clustering (SCENIC) analysis in snRNA-seq data

We used pyscenic (version 0.10.4) to perform single-cell regulatory network analysis. We performed the analysis by following the protocol steps described in SCENIC workflow^69^. We first determine TFs and their predicted target genes based on the correlation of gene expression across cells using “pyscenic grn” function with default parameters. And then use “pyscenic ctx” function to identify whether the predicted target genes have the corresponding motifs of TFs to refine target genes using default parameters with the database file “mm10_refseq-r80_10kb_up_and_down_tss. mc9nr. feather” and the motif information file “motifs-v9-nr.mgi-m0.001-o0.0.tbl”. The AUCell analysis was further performed using “pyscenic aucell” function with parameters “rank_threshold” 5000, “auc_threshold” 0.05 and “nes_threshold” 3. Identified regulons from pyscenic were further selected based on the average AUCell score across cells >0.02 and the number of genes in each regulon >10. Differential regulons were selected through visual inspection on the heatmap plot of normalized AUCell scores across cells. The regulatory network centered on transcription-related factors was screened out and visualized by Cytoscape.

### Generation of GDF11 knockout (GDF11^KO^) Neuro-2a cell line

The pX459 plasmid (pX459-U6-sgRNA-CMV-Cas9-T2A-PuroR) was used as previously described. The pX459 plasmid consists of three components, including Cas9 nuclease, puromycin acetyltransferase (PuroR) and a 20nt target-specific guide RNA (gRNA) designed for maximum knockout efficiency. The two GDF11-specific gRNA sequences were designed using sgRNA designer website (https://chopchop.cbu.uib.no/) as indicated below:

5’-GCCGAAGGTACACCCACAGT-3’; 5’-GAACCGGGTAAGGTAGCTTG-3’.

Two pX459 plasmids, each including different GDF11 sgRNA, were co-transfected into Neuro-2a cells using transfectamine 2000 (Thermo Fisher Scientific) according to the manufacturer’s protocol with appropriate cell confluence (> 50%) after plating. Different concentrations of puromycin were used to treat wild-type Neuro-2a cells and the lowest concentration of puromycin to kill Neuro-2a cells after 4 days’ treatment was 3 μg/ml. This concentration was subsequently used for selecting and enriching the PuroR antibiotic-resistant transfected Neuro-2a cells. At 48 h post-transfection, the culture media was removed by washing the cells with 0.01 M PBS and selection medium (complete medium containing 3 μg/ml of puromycin) was added for screening the cells. The transfected cells were cultured for 4 days with selection medium and the cells transfected successfully with pX459 plasmids survived. No gRNA but only Cas9 transfection was used as another control in addition to WT Neuro-2a cells. The GDF11^KO^ cell clone was screened out through single clone expansion protocol: the survived cells were counted and diluted with complete medium ensuring one single cell per 100 μl culture medium by cell counting. The cells were subsequently seeded into 96-well plates with 100 μl per well. After culture for approximately 14 days, cells in each well grown from a single cell thus was one clone which was verified by PCR, RT-PCR, WB and/or immunofluorescence either as a GDF11^KO^ or WT Neuro-2a clone. Three independent GDF11^KO^ cell clones were obtained. The confirmed GDF11^KO^ cells or WT Neuro-2a cells were cultured in routine method and were recorded as days in vitro (DIV), and were passaged every 3 days. We estimated that GDF11^KO^ cells underwent about two cell divisions within each passage in our experimental condition.

### Verification of knockout efficiency of GDF11KO Neuro-2a cell line

Genomic DNA was extracted from GDF11 knock-out (GDF11^KO^) Neuro-2a cells using a TIANamp Genomic DNA Kit (TIANGEN). Primers were designed according to the two GDF11-specific gRNA target sites with primer F (5’-CGCAGAATGGGGAGTCAGAA-3’) and primer R (5’- ATCCTCCACCACCACTTCAG-3’). PCR was performed to determine the knockout efficiency by analyzing different size bands on 1.2% agarose gel. PCR product of Neuro-2a cells was 514 bp for the GDF11^KO^ and 992 bp for the wild-type. For in vitro GDF11^KO^ experiments, the individual data points were obtained from three independent clones.

### Bulk RNA-seq library preparation

GDF11^KO^ and WT Neuro-2a cells cultured over 65 DIV were performed to bulk RNA-seq. Bulk RNA-seq libraries were prepared as we described previously^70^. In brief, total RNA from three mixed clones of GDF11^KO^ and WT Neuro-2a cells (*n* = 3 per group) was purified via RNA Isolation Kit (Vazyme). RNA-seq libraries were generated with NEBNext Ultra Directional RNA Library PrepKit (NEB, USA). Each library was sequenced with NovaSeq 6000 (Illumina, San Diego, CA, USA).

### RNA-seq data analysis

All fastq files were passed through the quality control tool FASTQC and were mapped to the mouse genome (mm10) using tophat (v2.1.1). Differentially expressed gene (DEG) analysis was performed to compare GDF11^KO^ and WT Neuro-2a cells using the cuffdiff (v2.2.1). DEGs were defined as *P* value < 0.05 and absolute log_2_ ≥ 1 (fold change ≥2). Gene ontology (GO) enrichment of biological processes was performed using PANTHER (http://pantherdb.org/).

### ChIP-qPCR

ChIP-qPCR experiments were performed as previously described^71^ with slight modifications. Briefly, three clones of GDF11^KO^ and WT Neuro-2a cells were cross-linked for 10 min fixed in 1% formaldehyde and quenched with 125 mM glycine for 5 min at room temperature. Then, cells were lysed and sonicated until the size of DNA fragment was 150–300 bp. Sonicated chromatin was immunoprecipitated by incubation with Smad2 antibody (10 μg, Cell Signaling, 5339) or IgG isotype control (10 μg) overnight at 4 °C. Immunoprecipitated complexes were obtained using protein A/G beads (SANTA CRUZ). Subsequently, beads were washed one time sequentially with low-salt buffer, high-salt buffer, LiCl buffer, twice with TE buffer, and then resuspended in elution buffer (1% SDS, 100 mM NaHCO_3_). The DNA in the elutes was incubated with Proteinase K (1 mg/ml) overnight at 65 °C to reverse cross-linking. Next, DNA was purified and extracted using a TIANamp Genomic DNA Kit (TIANGEN, Cat.DP304) and subjected to qPCR analyses. ChIP-qPCR primer were designed according to the predicted binding motif between Smad2/3 and the promoter of p21 (http://jaspar.genereg.net/). Primers used in ChIP-qPCR are:

Mouse *p21*—F 5’-CAGGAAGAGGAAATGGGGTCT-3’;

Mouse *p21*—R 5’-TGATGGAAAAGCCCAGACTGC-3’.

### Plasmid constructs

Mouse GDF11 and GDF8 expression plasmids (pMs-GDF11-Flag-Myc or pMs-GDF8-Flag-Myc) with C-terminal 3Flag and Myc tag were designed and produced by Origene. Human GDF11 and GDF8 expression plasmids (phGDF11-Flag or phGDF8-Flag) with C-terminal 3Flag tag were designed and produced by Vigene Biosciences. To generate pMs-GDF11-eGFP-Flag construct, mouse GDF11 sequence was subcloned into vector pEGFP-N2 (Clontech) with C-terminal eGFP and 3Flag tag. All obtained plasmids were confirmed through sequencing.

### RNA isolation and real-time qPCR

Total RNA from tissues or cells was extracted with RNA Isolation Kit (Vazyme) and reverse transcribed into cDNA using the HiScript cDNA Synthesis kit (Vazyme) according to the manufacturer’s protocol. Quantitative real-time PCR was run using SYBR qPCR master mix (Vazyme) in a CFX96 Touch Thermal Cycler (Bio-rad). Every sample was run in triplicates. qPCR reactions were performed according to the manufacturer’s protocol. Primers were designed as follows:

*GDF11*—F 5’-CAAACTGCGGCTCAAGGAG-3’;

*GDF11*—R 5’-TGGGGCTGAAGTGGAAATGA-3’;

*p21*—F 5’-CAGGCACCATGTCCAATCCT-3’;

*p21*—R 5’-CGTCTCCGTGACGAAGTCAA-3’;

*β-actin*—F 5’-CGCAGCCACTGTCGAGT-3’;

*β-actin*—R 5’-CCCACGATGGAGGGGAATAC-3’.

### Immunofluorescence staining

For tissue immunofluorescence staining, after anaesthetized using 1.5% pentobarbital sodium, mice were perfused transcardially using ice-cold PBS (pH 7.4) followed by 4% paraformaldehyde (PFA). After 4 h post fixation in 4% PFA solution, mouse brains were cryoprotected in 30% sucrose for 2 days. Mouse brain coronal sections (12 μm) were cut using a microtome (Leica) and harvested on adhesion microscope slides and stored at −20 °C for further use.

For cell immunofluorescence staining, after plated on small round slides, Neuro-2a cells were fixed by 4% paraformaldehyde for 15 min followed by immunofluorescence staining procedure.

For immunofluorescence (IF) labelling, the standard process was performed quantitatively to detect the expression of various proteins. The primary antibodies used were mouse anti-GDF11 (1:200, R&D, MAB19581), rabbit anti-GDF11 (1:200, Abcam, ab124721), rabbit anti-GDF11 (1:200, NOVUS, NBP1-95888), guinea pig anti-NeuN (1:1000, Millipore, ABN90), rabbit anti-CaMKIIα (1:500, Abcam, ab131468), rabbit anti-GABA (1:500, Sigma, A2052), rabbit anti-Olig2(1:200, Millipore, AB9610), chicken anti-GFAP (1:1000, Millipore, AB5541), rabbit anti-Iba1 (1:500, WAKO, 019−19741), rabbit anti-p21 (1:200, Cell Signaling, 2947) and goat anti-DCX (1:1000, Santa Cruz, sc-8066). Alexa fluor 488 or Cy3 conjugated secondary antibodies were used (1:500, Jackson Laboratories). Specially, for GDF11 or CaMKIIα staining in brain section, a pretreatment in citrate antigen retrieval solution (pH 6.0) heated by microwave is necessary, then the sections were incubated for 48–72 h at 4 °C with anti-GDF11 or anti-CaMKIIα antibody. DAPI were used to stain the cellular nuclei. However, GDF11 staining in cultured cells did not need antigen retrieval. An Olympus Fluoview FV1000 confocal microscope was used to acquire fluorescent images.

### Immunohistochemical staining

Mouse brain section (12 μm) preparation was the same as the immunofluorescence protocol described above. The standard immunohistochemistry was performed. In brief, the brain sections were pretreated for 20 min in citrate antigen retrieval solution (pH 6.0) heated by microwave and then quenched for 5 min using 3% H_2_O_2._ Then, brain sections were blocked at 4 °C for 2 h in blocking buffer (5% donkey serum, 1% BSA in 0.01 M PBS, pH 7.4). The primary antibodies used were rabbit anti-GDF11 (1:200, NOVUS, NBP1-95888) and rabbit anti-CaMKIIα (1:500, Abcam, ab131468). HRP conjugated secondary antibodies (1:500, Jackson Laboratories), VECTA stain ABC kit (Cat. PK-6100) and DAB staining kits (Sigma, Cat. D4293, brown; Cat. D0426, black) were used following manufacturer’s instructions. An Olympus microscope (BX53) was used to acquire images.

### Western blot

To extract total protein, cells were washed in 0.01 M PBS and resuspend in protein lysis buffer (50 mM Tris‐HCl (pH 7.5), 5 mM EDTA-Na2, 1% SDS, 1% Nonidet P40 Substitute (NP‐40 buffer), 0.5% sodium deoxycholate, 3.69% CHAPS (Biosharp), 1% Triton X-100, 50 mM NaCl) supplemented with 1 mM DTT, phosphatase inhibitors (Biotool), MG132 (carbobenzoxy-Leu-Leu-leucinal; Sigma) and protease inhibitors (Roche). After cells were homogenized and maintained on ice for 30 min, samples were centrifuged at 1000×*g* for 5 min at 4 °C, and the supernatants were collected. For tissue protein extraction, after a quick perfusion through the heart with ice-cold 0.01 M PBS (pH 7.4), mouse tissues were collected and homogenized in protein lysis buffer using a hand-held homogenizer on ice. After left on ice for 30 min, the tissue homogenates were centrifuged at 1000 × *g* for 5 min at 4 °C, and the supernatants were collected.

Protein concentrations were measured using BCA protein assay Kit (Beyotime). In total, 10–40 μg protein for each lane was analyzed on a 10% or 12% SDS–PAGE gel and wet transferred for western blot (WB) analysis. The membrane used for WB was PVDF (0.2 μm, Millipore). The protein loading buffer (FUDE China, Cat. FD002) contained reducing agents DTT and denaturant agents LDS (lithium dodecyl sulfate), and the samples were heated at 95 °C for 5 min before loading on the gel. The primary antibodies used were mouse anti-GDF11 (1:5000, R&D, MAB19581), mouse anti-GDF11 (1:2000, Santa Cruz, sc-81952), rabbit anti-GDF11 (1:2000, Abcam, ab124721), mouse anti-Myc (1:1000, Cell Signaling, 2276), mouse anti-β-actin (1:10,000, Sigma, A5441), rabbit anti-pSmad2 (1:1000, Cell Signaling, 3108), rabbit anti-Smad2 (1:2000, Cell Signaling, 5339), rabbit anti-Smad3 (1:2000, Cell Signaling, 9513) and rabbit anti-Tmem159 (1:1000, Biorbyt, orb351361). Secondary antibodies (Jackson Laboratories) conjugated with horseradish peroxidase (HRP) were used at the dilution of 1:10,000. All the bands were quantified using ImageJ.

### Electron microscopy

#### Transmission electron microscopy (TEM)

Three clones of GDF11^KO^ and WT Neuro-2a cells were fixed with 4% formaldehyde and 2.5% glutaraldehyde in 0.1 M phosphate buffer (pH 7.4) and scraped down with a cell scraper. Cells were post-fixed overnight with 4% formaldehyde and 2.5% glutaraldehyde in 0.1 M phosphate buffer (pH 7.4). After washed three times with 0.1 M phosphate buffer (pH 7.4), cells were fixed using 1% osmium tetra-oxide (OsO4) for 60 min at room temperature. The cells were subsequently rinsed twice with water for 10 min and then stained for 30 min at room temperature using 2% uranyl acetate. The cells were dehydrated in upgraded ethanol series: 50%, 70%, 90%, 100% and 100% followed by acetone twice and finally infiltrated in graded Epon series: 50% and 100%. The cells were embedded with 100% Epon at 37 °C for 24 h and then resin polymerization was performed at 60 °C in the dry oven for 48 h. Areas of interest region were cut into serial ultrathin sections with an ultramicrotome (Germany). After mounted on mesh grids (5–7 sections per grid), ultrathin sections were observed under a Tecnai G2 Spirit 120 kV Transmission Electron Microscope (TEM, Thermo FEI).

#### Immuno-electron microscopy (Immuno-EM)

For pre-embedding immuno-gold TEM, after anaesthetized using 1.5% pentobarbital sodium, wild-type (WT) male mice aged 3 M were perfused transcardially using ice-cold PBS (pH 7.4) followed by 0.1% glutaraldehyde, 4% formaldehyde in 0.1 M phosphate buffer (pH 7.4). The mouse brain was dissected quickly and post-fixed overnight with 0.1% glutaraldehyde, 4% formaldehyde in 0.1 M phosphate buffer (pH 7.4). Next, mouse brain coronal sections (50 μm) were cut using oscillating microtome (Leica VT1000S) in the 0.1 M phosphate buffer (pH 7.4) and post-fixed for 2 h in 0.1% glutaraldehyde, 4% formaldehyde in 0.1 M phosphate buffer (pH 7.4). After washed with PBS (pH 7.4), brain sections were incubated with 50 mM glycine in 0.1 M PBS (pH 7.4) for 30 min. Then, brain sections were permeabilized with 0.01% Triton X−100 in 0.1 M PBS for 15 min before blocking the brain sections with the block solution containing 0.1% BSA-c (Aurion) in 0.1 M PBS (pH 7.4). The sections were incubated in the anti-GDF11 antibody (R&D, MAB19581, 1:100) overnight at 4 °C followed by several times of rinse, and the sections were then incubated in the secondary antibody (Nanogold® -IgG Goat anti-mouse, Nanoprobes, 1:100) overnight. After washing in 0.1 M PB (pH 7.4), brain sections were post-fixed with 2.5% glutaraldehyde in 0.1 M PB and treated with the silver enhancement kit (Nanoprobes) according to the manufacturer’s protocol. Then, the sections were treated in 1% OsO4 for 60 min and in 2% uranyl acetate for 30 min. After gradient dehydration, brain sections were embedded in Epon and ultrathin sections were then observed under a Tecnai G2 Spirit 120 kV Transmission Electron Microscope (TEM, Thermo FEI).

### Quantification and statistical analysis

The experimenters were blinded to the grouping of images during image quantifications. Data analysis for quantification was performed by using ImageJ and Prism 8 (GraphPad Software). Image processing was performed with FV10-ASW 4.2 Viewer, PS (Adobe Photoshop CC 2018) and AI (Adobe Illustrator CC 2018). Data are presented as mean ± SEM with difference considered significant when *P* < 0.05. *P < 0.05, **P < 0.01, and “ns” indicates not significant.

### Reporting summary

Further information on research design is available in the Nature Portfolio Reporting Summary linked to this article.

### Supplementary information

Supplementary Information

Reporting Summary

### Source data

Source Data

## Data Availability

The RNA-seq data generated in this study have been deposited in the GEO database under accession code GSE167538. The snRNA-seq data generated in this study have been deposited in the China National GeneBank (CNGB) database under accession code CNGB: CNP0003558. The datasets are available from the corresponding author on request to Dr. Jing-Wei Zhao (jingweizhao@zju.edu.cn). Source data are provided with this paper.

## Code availability

Code for snRNA-seq analysis is available at https://github.com/changhuizhong/GitCode.git.
