# Supplementary material for: GDF11 slows excitatory neuronal senescence and brain ageing by repressing p21
Source: Nat Commun. 2023 Nov 17;14:7476. doi: 10.1038/s41467-023-43292-1 (PMC10656444; doi:10.1038/s41467-023-43292-1)
Supplement: Supplementary file 1 — Supplementary Information [file 41467_2023_43292_MOESM1_ESM.pdf]

GDF11 slows excitatory neuronal senescence and brain ageing by repressing p21

Supplementary Figure titles and legends

Supplementary Fig.1

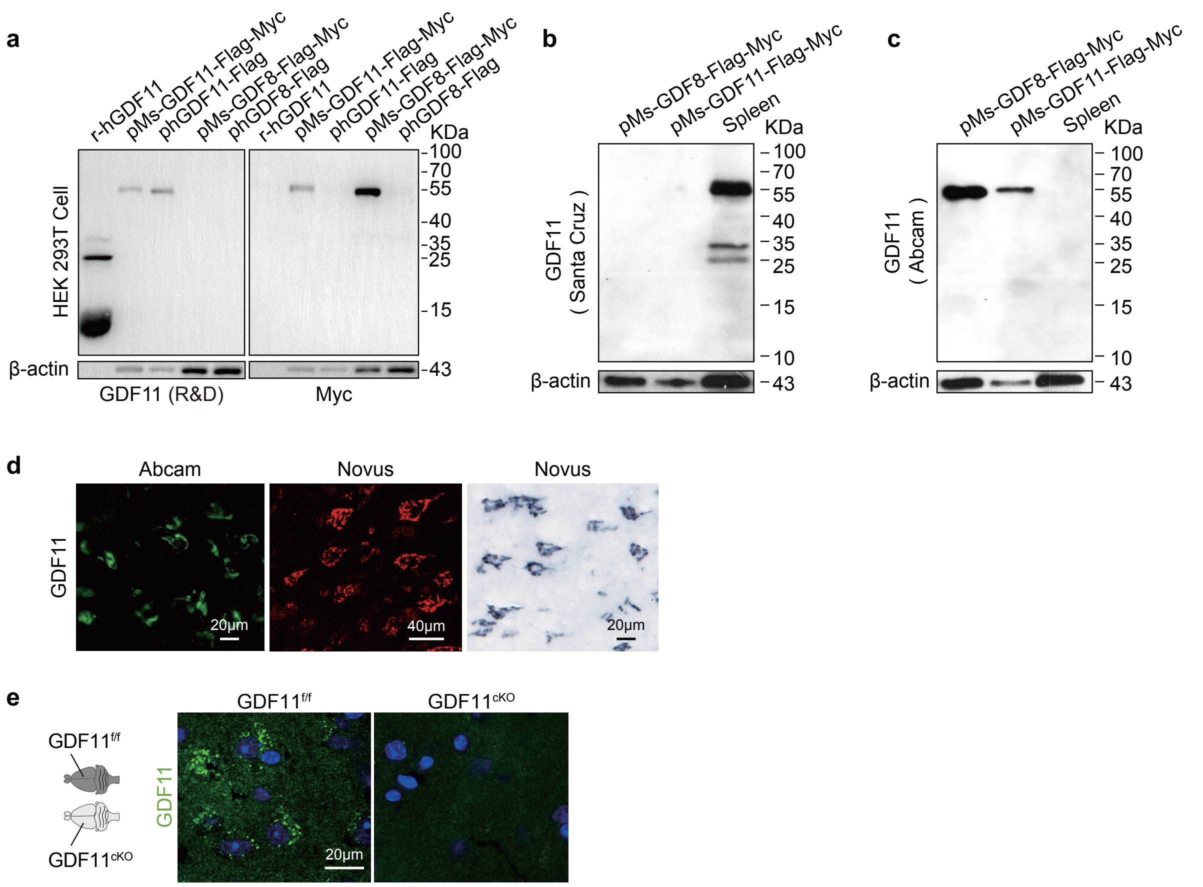

Supplementary Fig.1. Identification of a specific antibody for GDF11. a, Western blot of protein lysates from HEK 293T cells transfected with various tags fused with coding domain sequence (CDS) of mouse GDF11 or GDF8 DNA using antibodies of GDF11 (R&D, MAB19581, mouse anti-GDF11 antibody) or Myc, and the recombinant human mature GDF11 (rhGDF11) was used a positive control. Proteins were loaded in identical pattern, run in the same gel and were then immunoblotted in two separate blots. **b**, Representative Western blot images of total protein lysates from mouse spleen and HEK 293T cells transfected with various tags fused with the coding domain sequence (CDS) of mouse GDF11 or mouse GDF8 using anti-GDF11 antibody (Santa Cruz sc-81952), showing that this anti-GDF11 antibody detected neither GDF8 nor GDF11 protein in HEK 293 T cells and while detected multiple bands in spleen. **c**, Whole cell lysis of mouse spleen and HEK 293T cells transfected with mouse GDF8-Flag-Myc or mouse GDF11-Flag-Myc were immunoblotted with GDF11 antibody from Abcam (ab124721) showing this antibody cross-reacted with both GDF11 and GDF8 protein but failed to detect in the spleen, indicating this antibody is not specific to GDF11. **d,** Representative immunofluorescence images of the cerebral cortex of the mice aged 3M using anti-GDF11 antibody from Abcam (ab124721, green, left, scale bar, 20 μm) or NOVUS (NBP1-95888, EPR4567(2)) by immunofluorescence (red, middle, scale bar, 40 μm) or

immunohistochemistry-Nickel-DAB staining (black, right, scale bar, 20 μm). **e,** Representative immunofluorescence images of the cerebral cortex using the anti-GDF11 Ab1 (R&D, MAB19581) in $GDF11^{f/f}$ or $GDF11^{cKO}$ mice at age of 3M (n = 3 mice). GDF11 was selectively deleted in $CaMKII\alpha^{+}$ neurons through Cre/Loxp system in $GDF11^{cKO}$ mice, indicating that the R&D MAB19581 is a specific antibody to GDF11 (scale bar, 20 μm).

Supplementary Fig.2

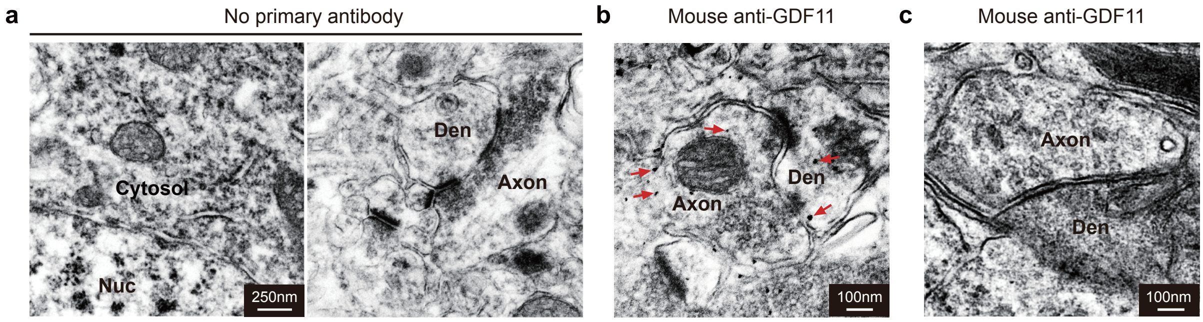

Supplementary Fig.2. Specificity verification of the immuno-EM labelling of GDF11. a, Using labelling protocol identical to **B** but without adding primary antibody, mouse anti-GDF11 antibody (R&D MAB19581), while the secondary antibody conjugated with nanogold particles was used, immuno-EM images of the cerebral cortex of the WT mice aged 3M show no nanogold particles labelling (negative control). Scale bar, 250 nm. **b**, In comparison with **a**, when anti-GDF11 antibody (R&D MAB19581) was used followed by the secondary antibody conjugated with nanogold particles, immuno-EM images reveal the localization of GDF11 (many black dots are GDF11 labelled and only some examples are indicated with red arrows) in the cerebral cortex of the WT mice aged 3M. The axon (Axon) and dendrite (Den) of an excitatory neuron with thickening postsynaptic membrane were positive. Scale bar, 250 nm. **c**, Representative images of immuno-EM of GDF11 labelled with nanogold particles, and there are no GDF11 labelled black dots at the inhibitory synapse of cerebral cortex in the mice aged 3M (n = 3 mice). Scale bar, 100 nm. Nuc, nucleus; Den, dendrite.

Supplementary Fig.3

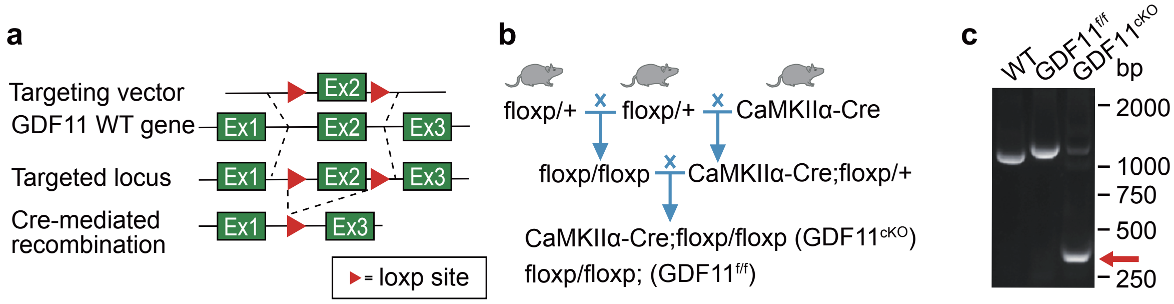

Supplementary Fig.3. Genetic strategy for generation of GDF11$^{f/f}$ mice and GDF11^{cKO} mice. **a** and **b**, Genetic strategy for generation of GDF11$^{f/f}$ mice (**a**) and GDF11^{cKO} mice (**b**) to selectively delete GDF11 in CaMKIIα^{+} neurons through Cre/Loxp system. Ex, exon. **c**, PCR of the cerebral cortex tissue genomes verified successful establishment of GDF11^{cKO} mice. The cerebral cortex tissue was selectively dissected from the brain of WT, GDF11$^{f/f}$ and GDF11^{cKO} mice. Red arrow indicates the knockout band in the GDF11^{cKO} mice.

Supplementary Fig.4

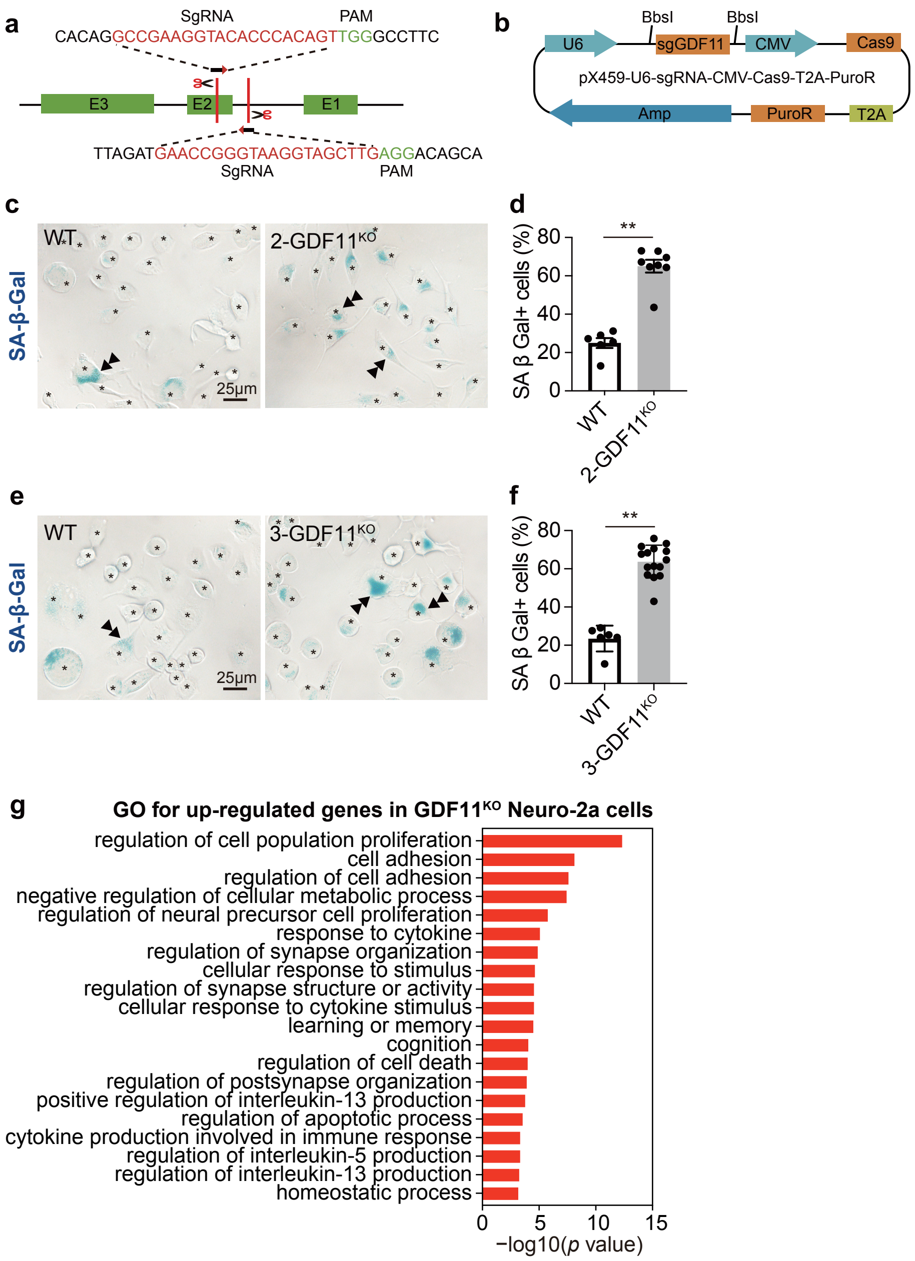

Supplementary Fig.4. Knocking-out GDF11 in Neuro-2a cells increases the proportion of SA-β-Gal⁺ cells. **a** and **b**, Genetic strategy for knocking out GDF11 in Neuro-2a cells using CRISPR/Cas9 technique (**a**) and the pX459 plasmid with target SgRNA of GDF11 (**b**). **c-f**, Representative images (**c** and **e**) and quantification of the senescence associated β-Galactosidase staining ($SA\text{-}\beta\text{-}Gal^{+}$ cells displayed in blue) cells in $2\text{-}GDF11^{KO}$ (**d**, $2\text{-}GDF11^{KO}$, n = 8; WT, n = 6 fields) or $3\text{-}GDF11^{KO}$ (**f**, $3\text{-}GDF11^{KO}$, n = 15; WT, n = 6 fields) and WT Neuro-2a cells. All cells are marked with star. Some $SA\text{-}\beta\text{-}Gal^{+}$ cells are indicated with double arrowheads. **g**, Bulk RNA-seq gene ontology (GO) analysis revealed the 20 enriched cellular senescence and aging related biological processes upregulated by GDF11 deletion in Neuro-2a cells.

c and **e**, Scale bar, 25 μm. **d** ($p < 0.0001$) and **f** ($p < 0.0001$), data are presented as mean ± SEM. *$p < 0.05$, **$p < 0.01$; unpaired two-tailed t test.

Supplementary Fig.5

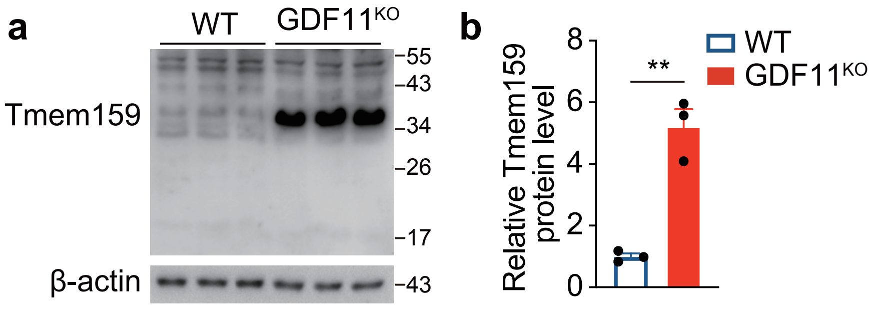

Supplementary Fig.5. *In vitro* loss of GDF11 up-regulates Tmem159. a and **b**, Representative images (**a**) and quantification by densitometry of Western blot analysis of Tmem159 (**b**) in the total protein extracted from the $GDF11^{KO}$ and WT Neuro-2a cells (n = 3 biological repeats/group). **b** ($p = 0.0019$), data are presented as mean ± SEM. *$p < 0.05$, **$p < 0.01$; unpaired two-tailed t test.

Supplementary Fig.6

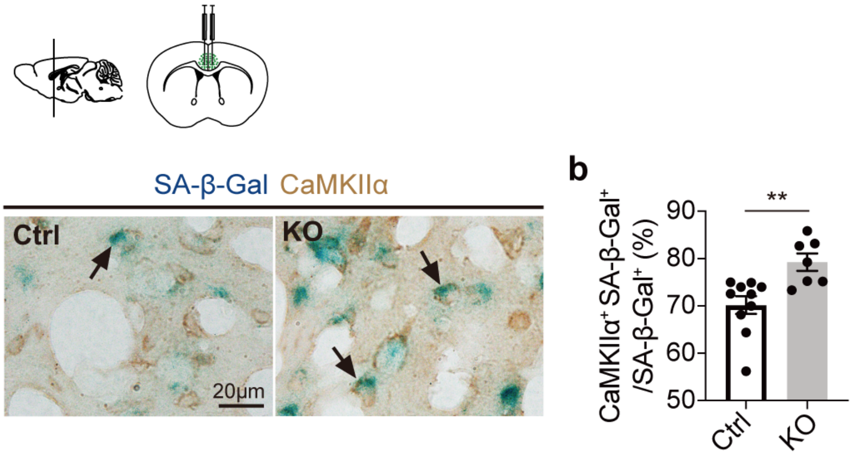

Supplementary Fig.6. *In vivo* selective deletion of GDF11 in ENs of the cingulate cortex promotes cellular senescence in ENs. a and **b**, Representative images (**a**) and quantification (**b**) of double labelling of SA-β-Gal staining (blue) and immunohistochemical staining of CaMKIIα (brown) in the cingulate cortex of the $GDF11^{f/f}$ mice which received a focal injection of AAV9-CaMKIIα-GFP virus (Ctrl, $GDF11^{f/f}$, left, n = 10) or AAV9-CaMKIIα-Cre-P2A-GFP virus (KO, $fGDF11^{cKO}$, right, n = 7) at age of 2-3M and survived for two more months and were perfused at age of 4-5M.

a, scale bars, as shown on the images, 20 μm. **b** (p = 0.0045), data are presented as mean ± SEM. *p < 0.05, **p < 0.01; unpaired two-tailed t test.

Supplementary Fig.7

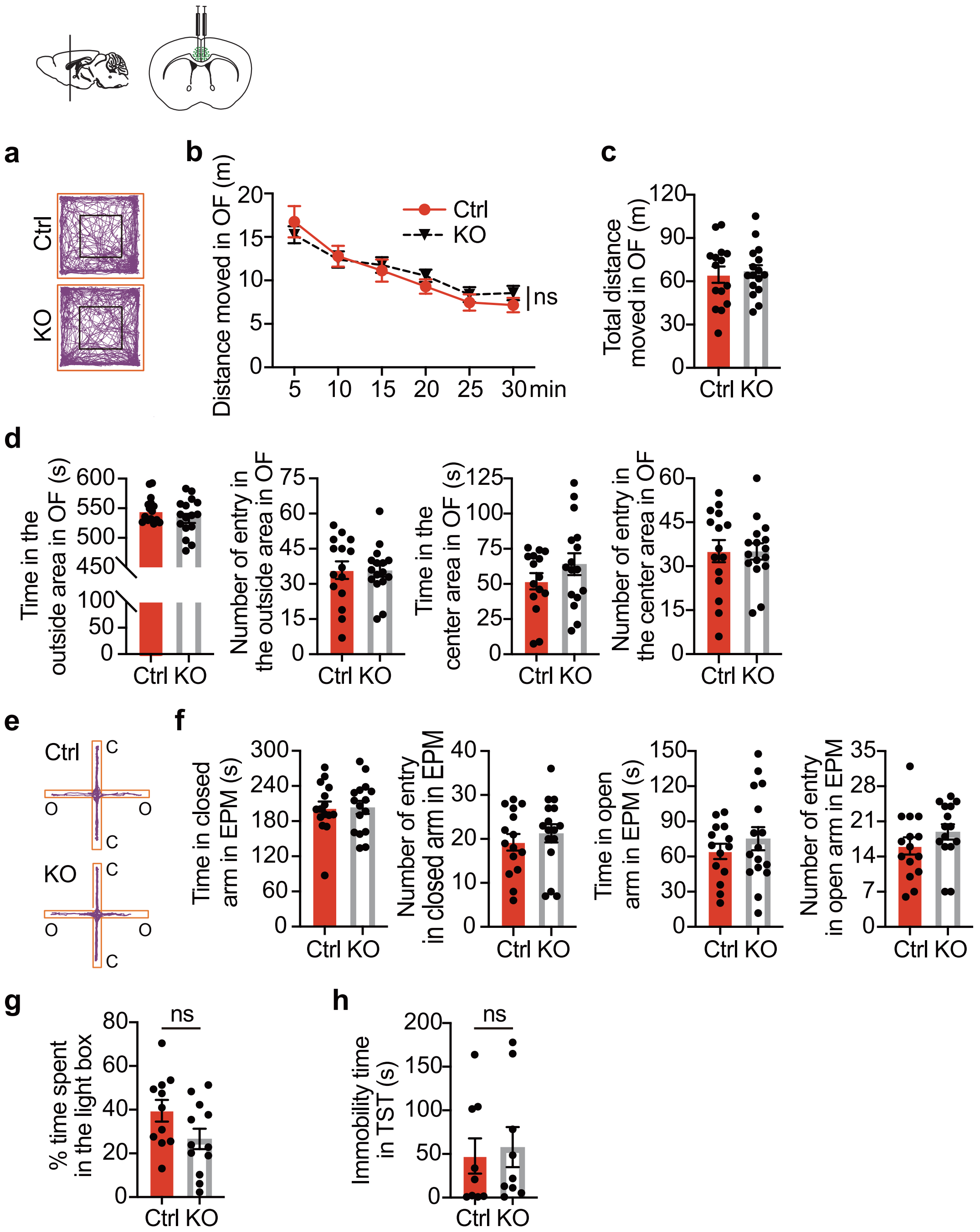

Supplementary Fig.7. *In vivo* selective deletion of GDF11 in ENs of the cingulate cortex does not affect locomotion, anxiety-like behavior, aversive stimuli-driven memory and learning and despair behavior. The cingulate cortex of the GDF11$^{f/f}$ mice received a focal injection of AAV9-CaMKIIα-Cre-P2A-GFP virus (KO, fGDF11^{cKO}) or AAV9-CaMKIIα-GFP virus (Ctrl, GDF11$^{f/f}$) at age of 2-3M and survived for two more months and tests were performed when the mice were at age of 4-5M. **a**, Track diagrams in the open filed (OF) test between the KO and Ctrl groups. **b**, The line chart of moved distance during different time periods in the open filed of the KO and Ctrl groups (KO, n = 16; Ctrl, n = 15 mice). **c**, The total moved distance during the 30 min in the open filed of the KO and Ctrl groups (KO, n = 16; Ctrl, n = 15 mice). **d**, The time spent and the number

of entries in the outside or the central area of the open field in the KO and Ctrl groups. (KO, $n = 16$; Ctrl, $n = 15$ mice). **e**, Track diagrams in the elevated plus maze (EPM) test between the KO and Ctrl groups. **f**, Analysis of the time and the number of entries in the open or closed arm of the EPM test in the KO and Ctrl groups (KO, $n = 16$; Ctrl, $n = 15$ mice). **g**, The percentage of time spent in the light area during the passive avoidance test (PAT, light-dark box test) in the KO and Ctrl groups (KO, $n = 12$; Ctrl, $n = 11$ mice). **h**, The immobility time in the tail suspension test (TST) between the KO and Ctrl groups (KO, $n = 9$; Ctrl, $n = 9$ mice).

Data are presented as mean ± SEM. *$p < 0.05$, **$p < 0.01$ and “ns” indicates not significant. **b** (5: Ctrl versus KO, $p = 0.8914$; 10: Ctrl versus KO, $p > 0.9999$; 15: Ctrl versus KO, $p = 0.9982$; 20: Ctrl versus KO, $p = 0.9545$; 25: Ctrl versus KO, $p = 0.9905$; 30: Ctrl versus KO, $p = 0.9282$), two-way ANOVA with post Sidak's multiple comparisons test; **c** ($p = 0.7460$), **d** (left1: $p = 0.2204$; left2: $p = 0.9681$; left3: $p = 0.2204$; left4: $p = 0.9765$), **f** (left1: $p = 0.9422$; left2: $p = 0.4738$; left3: p = 0.0.3798; left4: $p = 0.2372$;), **g** ($p = 0.0753$) and **h** ($p = 0.7394$), unpaired two-tailed t test.

Supplementary Fig.8

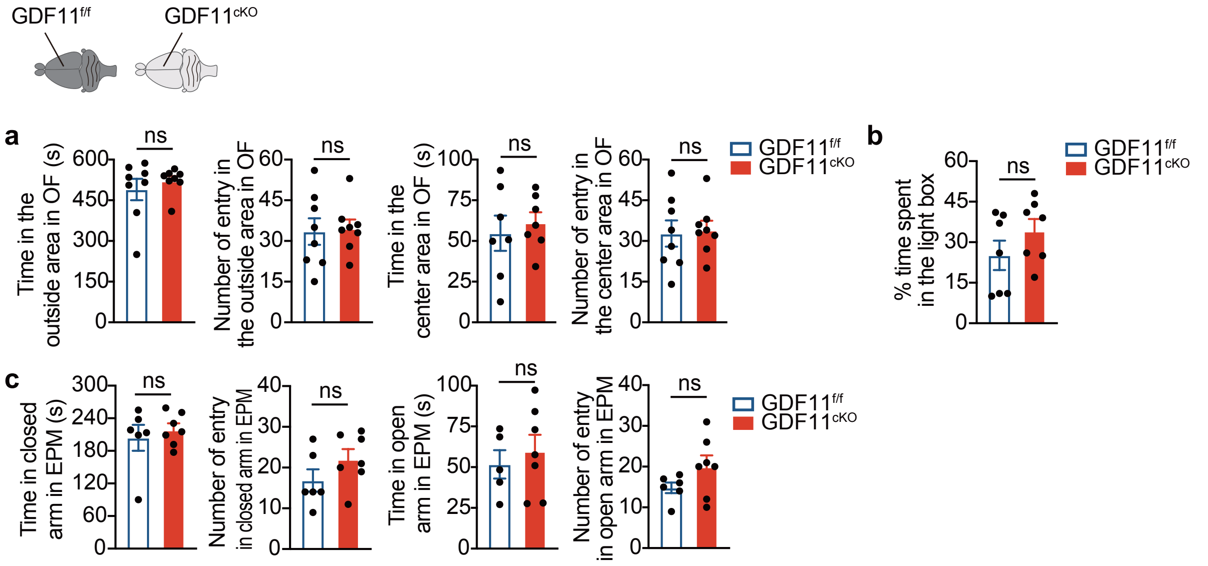

Supplementary Fig.8. *In vivo* selective deletion of GDF11 in ENs in the CNS does not affect locomotion, aversive stimuli-driven memory and learning and anxiety-like behaviors. a, The time spent and the number of entries in the outside or the central area of the open field in the $GDF11^{cKO}$ and $GDF11^{f/f}$ mice aged 10M ($GDF11^{cKO}$, n= 10; $GDF11^{f/f}$, n = 6 mice). **b**, The percentage of time spent in the light area during the passive avoidance test (PAT, light-dark box test) in the $GDF11^{cKO}$ and $GDF11^{f/f}$ mice aged 10M ($GDF11^{cKO}$, n= 10; $GDF11^{f/f}$, n = 7 mice). **c**, Analysis of the time and the number of entries in the open or closed arm of the elevated plus maze (EPM) in the $GDF11^{cKO}$ and $GDF11^{f/f}$ mice aged 10M ($GDF11^{cKO}$, n= 10; $GDF11^{f/f}$, n = 7 mice).

a (left1: p = 0.4642; left2: p = 0.8343; left3: p = 0.6112; left4: p = 0.8182), **b** (p = 0.2148), **c** (left1: p = 0.5553; left2: p = 0.1730; left3: p = 0.5770; left4: p = 0.1390), Data are presented as mean ± SEM. *p < 0.05, **p < 0.01 and “ns” indicates not significant, unpaired two-tailed t test.

Supplementary Fig.9

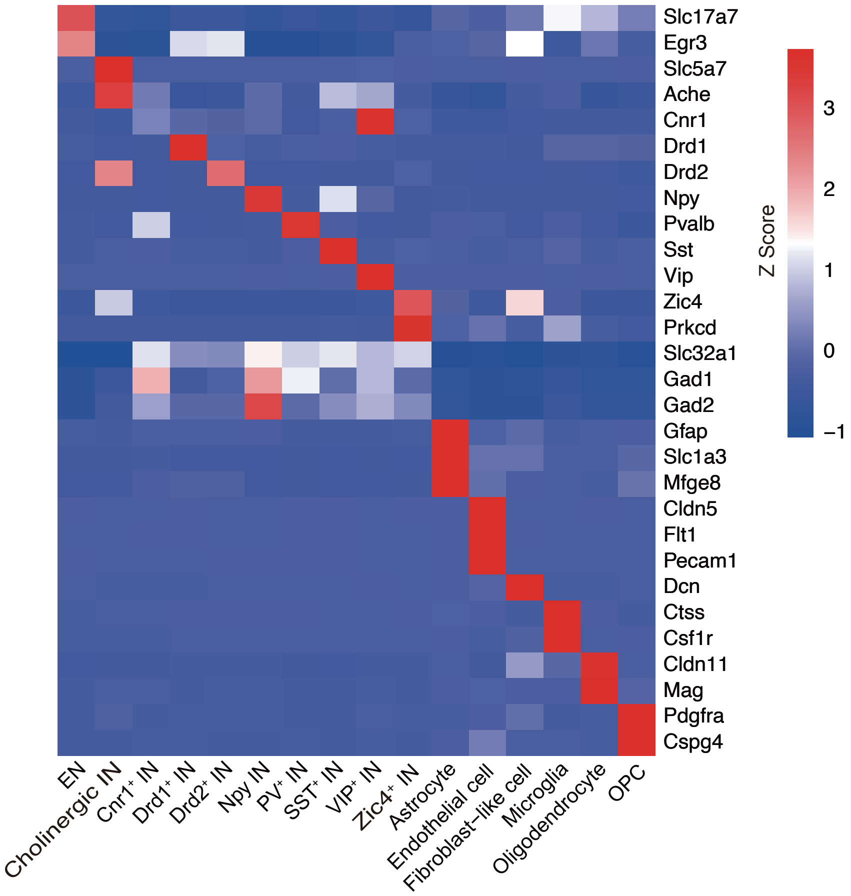

Supplementary Fig.9. Clustering of 16 different cell types in snRNA-seq by the transcription of marker genes. The single nuclei were obtained from the cingulate cortex of the "KO" mice and the "Ctrl" mice aged 4-5M. Heatmap showing the average expression and the frequency of expression of the marker genes used to identify the cell type among cells of various lineages in our snRNA-seq of the adult mouse cortex.

Supplementary Fig.10

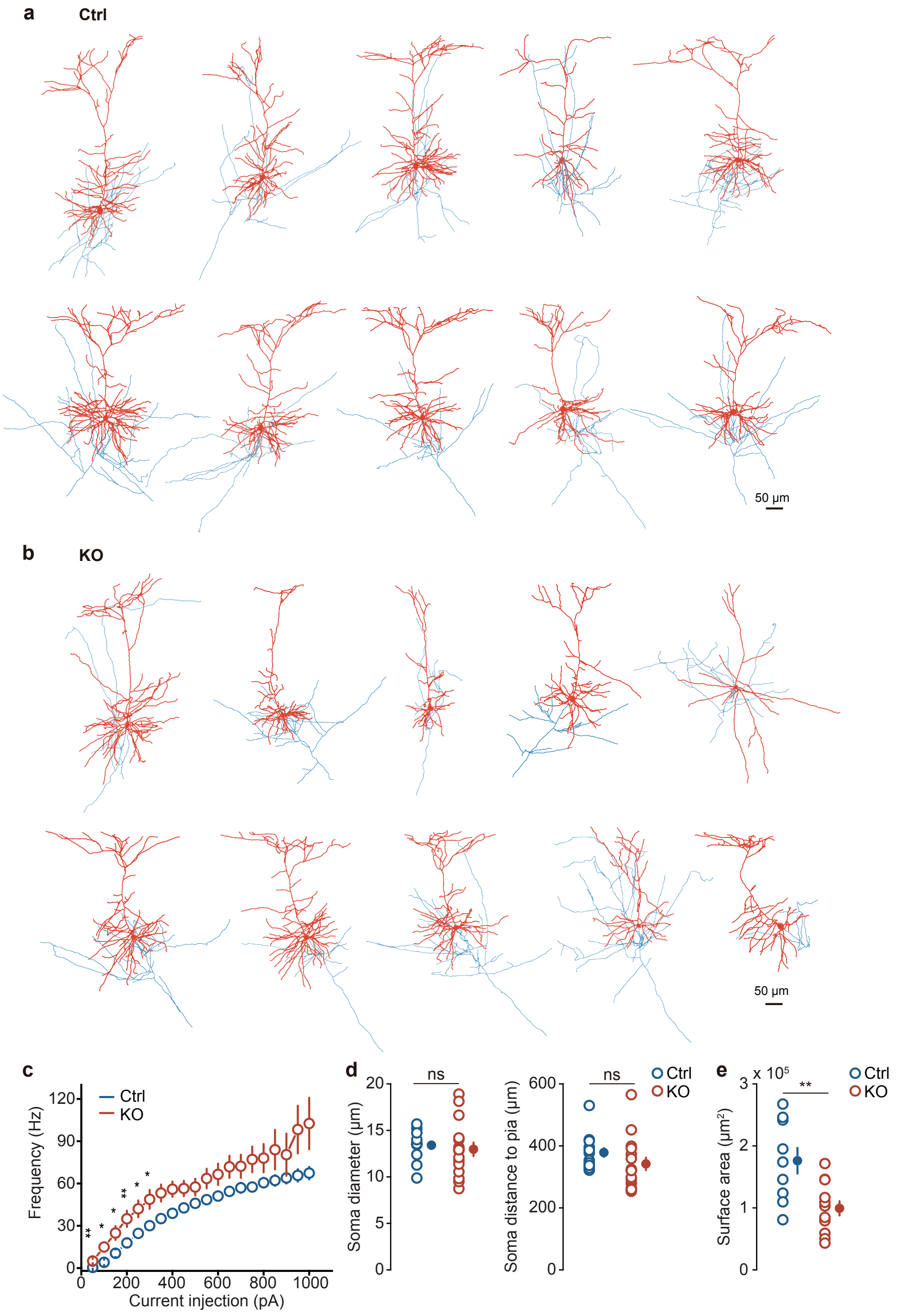

Supplementary Fig.10. 3D-reconstruction of representative ENs in the cingulate cortex of the control or fGDF11^{cKO} mice. Biocytin in the patch pipettes allowed post hoc streptavidin staining, visualization and 3D reconstruction of the labelled morphological structure of the ENs in the cingulate cortex of the "KO" mice and the "Ctrl" mice aged 4-5M. **a** and **b**, 3D-reconstruction of 10 other ENs in the cingulate cortex of fGDF11^{cKO} (KO, **b**) and control (Ctrl, **a**) mice. Dendrites and soma are represented in red while the axons are in blue. **c**, Plots of the AP frequency as a function

of injected currents from cingulate cortex (Ctrl, n = 26 cells from 3 mice; KO, n = 19 cells from 3 mice). Only ENs with regular firing pattern were selected. Curves are color coded. **d**, Plots of the soma diameter (left, ctrl: 13.4 ± 0.5 vs. KO: 13.0 ± 0.7 μm, p = 0.647) and the soma location distance to the pia (right, ctrl: 379 ± 17 vs. KO: 342 ± 20 μm, p = 0.054) in the KO (n = 16 cells from 3 mice) and Ctrl (n = 12 cells from 3 mice) mice. **e**, Group plots of neuronal surface area (Ctrl: 1.76 ± 0.22 x 10^5 vs. KO: 0.99 ± 0.12 x 10^5 μm^2, p = 0.004) in the KO (n = 12 cells from 3 mice) and Ctrl (n = 9 cells from 3 mice) mice.

Scale bar, 50 μm in both **a** and **b**. Data are presented as mean ± SEM. *p < 0.05, **p < 0.01, and "ns" indicates not significant. c, d (soma diameter) and e, unpaired two-tailed t test; d (soma distance to pia), Mann-Whitney U test.

Supplementary Fig.11

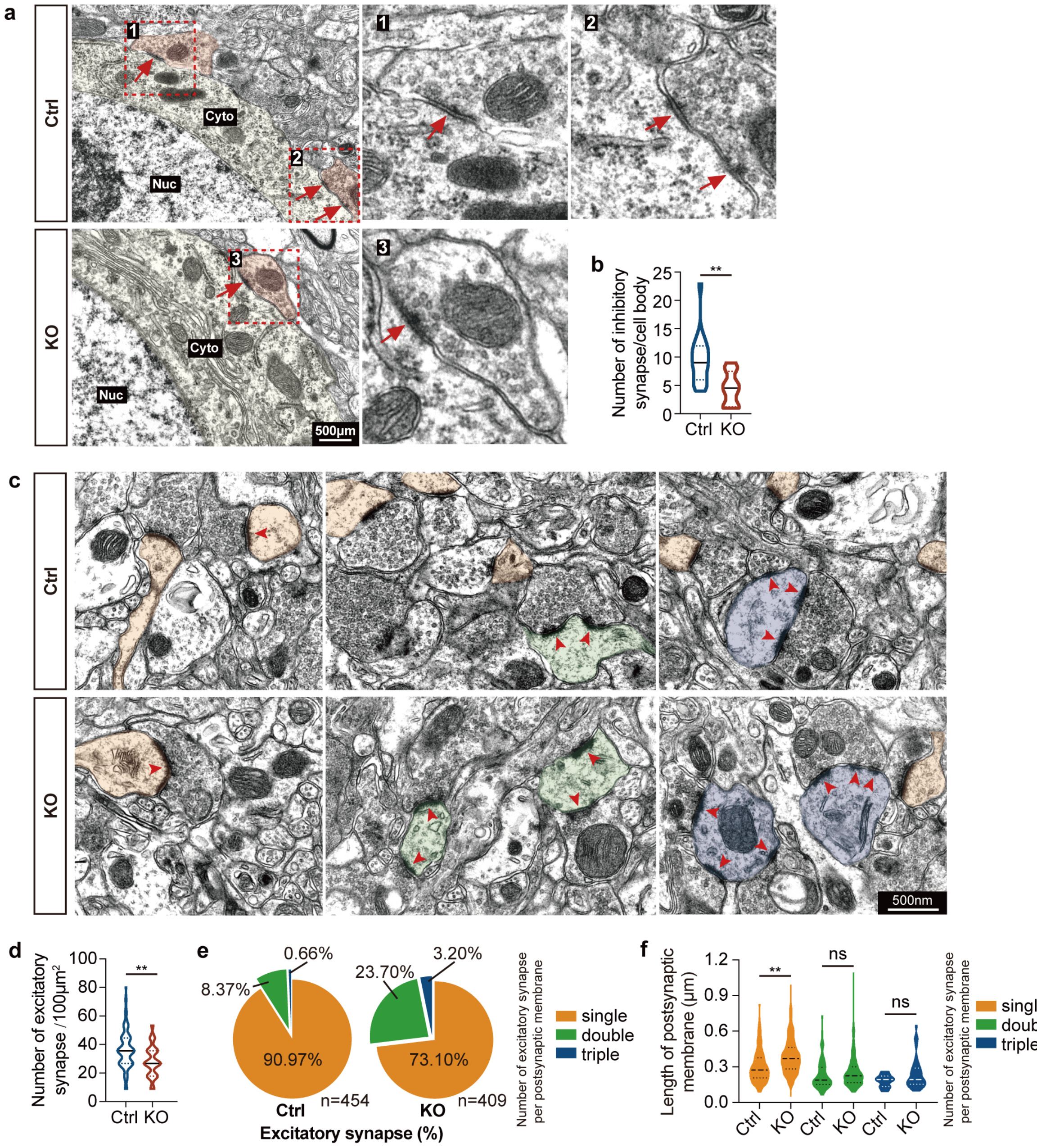

Supplementary Fig.11. ***In vivo*** **selective deletion of GDF11 in ENs reduces inhibitory synapse and increases excitatory synapse density. a** and **b**, Representative TEM micrographs (**a**) and quantification (**b**, KO, n=16; Ctrl, n=19 cells) of the number of inhibitory synapses per excitatory neuron body between KO and Ctrl group. The enlarged inhibitory synapses marked with number are showed on the right and indicated by arrows. Cyto, cytoplasm; Nuc, nucleus. **c**, Representative TEM micrographs of excitatory synapses between KO and Ctrl group. The excitatory synapses are indicated by arrows. Orange, single excitatory synapse per postsynaptic membrane; green, double excitatory synapses per postsynaptic membrane; blue, triple excitatory synapse per postsynaptic membrane. **d**, Quantification of the number of excitatory synapses per 100 μm^2 between KO and Ctrl group (KO, n=129; Ctrl, n=112 fields). **e**, The percentage of single or double or triple excitatory synapses per postsynaptic membrane between KO and Ctrl group. **f**,

Quantification of the length of postsynaptic membrane between KO and Ctrl group (KO single, n=299; Ctrl single, n=413; KO double, n=194; Ctrl double, n=76; KO triple, n=39; Ctrl triple, n=9 postsynaptic membrane).

Scale bars, as shown on the images, 500μm (**a**), 500nm (**c**). Data are presented as mean ± SEM. *$p < 0.05$, **$p < 0.01$ and "ns" indicates not significant. **b** ($p = 0.0003$), **d** ($p < 0.0001$) and **f** (single: $p < 0.0001$; double: $p = 0.1633$; triple: $p = 0.1899$), unpaired two-tailed t test.

Supplementary Fig.12

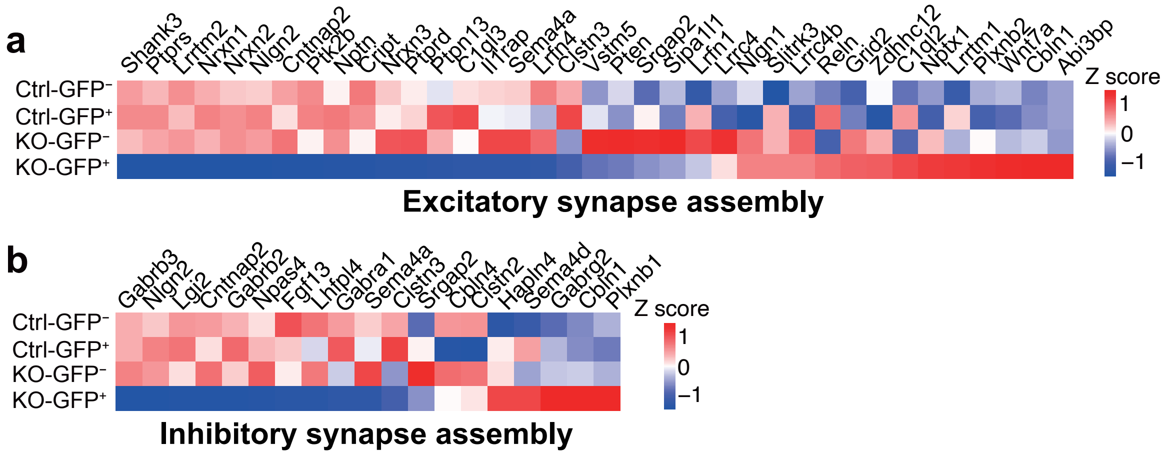

Supplementary Fig.12. *In vivo* selective deletion of GDF11 in the ENs affects the transcription of both the excitatory and the inhibitory synapse assembly related genes. a and **b**, Heatmap shows the average transcription of down-regulated and up-regulated excitatory synapse assembly (**a**) and inhibitory synapse assembly (**b**) related genes in snRNA-seq of KO-GFP$^+$, KO-GFP$^-$, Ctrl-GFP$^+$ and Ctrl-GFP$^-$ ENs.

Supplementary table title and legend

Supplementary Table 1

Information of Four GDF11 Antibodies

Company	Cat. Number	Epitope	Specificity	Application
R&D	MAB19581<br>Clone#743833	Asn299 - Ser407	GDF11	IF (√), WB (√)
Santa Cruz	sc-81952	N/A	No GDF11, No GDF8	IF (×), WB (√)
Abcam	ab124721<br>EPR4567(2)	aa350 - C - terminus	GDF11, GDF8	IF (√), WB (√)
Novus	NBP1-95888<br>EPR4567(2)	N/A	GDF11, GDF8	IF (√), WB (√)

Supplementary Table 1. Characterization of four commercially available anti-GDF11 antibodies. Summary of the information on the four anti-GDF11 antibodies from R&D, Santa Cruz, Abcam and NOVUS. Among four commercially available antibodies, the anti-GDF11 antibody from R&D System (MAB19581) is specific to GDF11 protein. The anti-GDF11 antibody from Santa Cruz did not detect either GDF11 or GDF8 protein, whereas the anti-GDF11 antibodies from Abcam and Novus did not distinguish between GDF11 and GDF8 proteins.
