# Supplementary material for: GDF11 slows excitatory neuronal senescence and brain ageing by repressing p21
Source: Nat Commun. 2023 Nov 17;14:7476. doi: 10.1038/s41467-023-43292-1 (PMC10656444; doi:10.1038/s41467-023-43292-1)
Supplement: Supplementary file 2 — Reporting Summary [file 41467_2023_43292_MOESM2_ESM.pdf]

nature portfolio

Corresponding author(s): Jing-Wei Zhao

Last updated by author(s): Oct 13, 2023

Reporting Summary

Nature Portfolio wishes to improve the reproducibility of the work that we publish. This form provides structure for consistency and transparency in reporting. For further information on Nature Portfolio policies, see our Editorial Policies and the Editorial Policy Checklist.

Statistics

For all statistical analyses, confirm that the following items are present in the figure legend, table legend, main text, or Methods section.

n/a	Confirmed	
☐	☒	The exact sample size (*n*) for each experimental group/condition, given as a discrete number and unit of measurement
☐	☒	A statement on whether measurements were taken from distinct samples or whether the same sample was measured repeatedly
☐	☒	The statistical test(s) used AND whether they are one- or two-sided<br>*Only common tests should be described solely by name; describe more complex techniques in the Methods section.*
☒	☐	A description of all covariates tested
☐	☒	A description of any assumptions or corrections, such as tests of normality and adjustment for multiple comparisons
☐	☒	A full description of the statistical parameters including central tendency (e.g. means) or other basic estimates (e.g. regression coefficient) AND variation (e.g. standard deviation) or associated estimates of uncertainty (e.g. confidence intervals)
☐	☒	For null hypothesis testing, the test statistic (e.g. F, t, r) with confidence intervals, effect sizes, degrees of freedom and P value noted<br>*Give P values as exact values whenever suitable.*
☒	☐	For Bayesian analysis, information on the choice of priors and Markov chain Monte Carlo settings
☒	☐	For hierarchical and complex designs, identification of the appropriate level for tests and full reporting of outcomes
☒	☐	Estimates of effect sizes (e.g. Cohen's d, Pearson's r), indicating how they were calculated

Our web collection on statistics for biologists contains articles on many of the points above.

Software and code

Policy information about availability of computer code

Data collection	CFX Manager software (Version 3.0)<br>Cuffdiff (v2.2.1) http://cole-trapnell-lab.github.io/cufflinks/cuffdiff/<br>Tophat (v2.1.1) http://ccb.jhu.edu/software/tophat/index.shtml<br>R (Version 3.6.0) https://www.r-project.org/
Data analysis	GraphPad Prism 8.0 https://www.graphpad.com/scientific-software/prism/<br>ImageJ https://imagej.nih.gov/ij/<br>Adobe Photoshop CC 2018<br>Adobe Illustrator CC 2018<br>FV10-ASW 4.2 Viewer<br>CFX Manager software (Version 3.0)<br>Cuffdiff (v2.2.1) http://cole-trapnell-lab.github.io/cufflinks/cuffdiff/<br>Tophat (v2.1.1) http://ccb.jhu.edu/software/tophat/index.shtml<br>R (Version 3.6.0) https://www.r-project.org/<br>PISA (v0.2) https://github.com/shiquan/PISA

For manuscripts utilizing custom algorithms or software that are central to the research but not yet described in published literature, software must be made available to editors and reviewers. We strongly encourage code deposition in a community repository (e.g. GitHub). See the Nature Portfolio guidelines for submitting code & software for further information.

Data

Policy information about availability of data

All manuscripts must include a data availability statement. This statement should provide the following information, where applicable:

- Accession codes, unique identifiers, or web links for publicly available datasets
- A description of any restrictions on data availability
- For clinical datasets or third party data, please ensure that the statement adheres to our policy

The RNA-seq data generated in this study have been deposited in the GEO database under accession code GSE167538 [https://www.ncbi.nlm.nih.gov/geo/query/acc.cgi?acc=GSE167538].

The snRNA-seq data generated in this study have been deposited in the China National GeneBank (CNGB) database under accession code CNGB: CNP0003558 [https://db.cngb.org/search/project/CNP0003558/].

Code for snRNA-seq analysis [https://github.com/changhuizhong/GitCode.git] is available.

The statistical data generated in this study are provided in the Source Data file.

The datasets generated during the current study are available from the corresponding author.

Human research participants

Policy information about studies involving human research participants and Sex and Gender in Research.

Reporting on sex and gender	Sex was not considered in this study design and methods.
Population characteristics	Six human samples were used in this study to avoid the possible variation. Two patients, aged 23 (female) and 24 (male) years respectively, who were diagnosed with intractable epilepsy and the focus of epilepsy had to be removed surgically. In addition, the frontal lobe tissues were obtained from other four patients, aged 23 (male), 52 (male), 54 (female) and 60 (male) years, respectively, who were suffering acute traumatic brain injury.
Recruitment	Human brain tissues were obtained from the First Affiliated Hospital, Zhejiang University School of Medicine.
Ethics oversight	The studies were approved by Medical Ethics Review Committee of Zhejiang University School of Medicine (ETHICS number : IIT20220453B).

Note that full information on the approval of the study protocol must also be provided in the manuscript.

Field-specific reporting

Please select the one below that is the best fit for your research. If you are not sure, read the appropriate sections before making your selection.

[x] Life sciences   [ ] Behavioural & social sciences   [ ] Ecological, evolutionary & environmental sciences

For a reference copy of the document with all sections, see nature.com/documents/nr-reporting-summary-flat.pdf

Life sciences study design

All studies must disclose on these points even when the disclosure is negative.

Sample size	No sample size calculation was performed to predetermine sample size. Sample size was determined according to the previous experimental observations and guidance in the field:<br>1) Cell stem cell 25, 514-530 e518, (2019). https://pubmed.ncbi.nlm.nih.gov/31543366/<br>2) Nat Aging 3, 213-228, (2023). https://pubmed.ncbi.nlm.nih.gov/37118117/
Data exclusions	No data were excluded from the analyses.
Replication	All the data in this study are representative of at least three experiments, unless otherwise statements. Experiments were performed independently.
Randomization	Samples/organisms/participants were allocated into experimental groups randomly.
Blinding	The investigators were blinded to group allocation during data collection and analysis.

Reporting for specific materials, systems and methods

We require information from authors about some types of materials, experimental systems and methods used in many studies. Here, indicate whether each material, system or method listed is relevant to your study. If you are not sure if a list item applies to your research, read the appropriate section before selecting a response.

Materials & experimental systems

n/a	Involved in the study
☐	☒ Antibodies
☐	☒ Eukaryotic cell lines
☒	☐ Palaeontology and archaeology
☐	☒ Animals and other organisms
☒	☐ Clinical data
☒	☐ Dual use research of concern

Methods

n/a	Involved in the study
☒	☐ ChIP-seq
☒	☐ Flow cytometry
☒	☐ MRI-based neuroimaging

Antibodies

Antibodies used

1) Mouse anti-GDF11, R&D Systems Cat#MAB19581, 1:200; RRID: AB_2877166
2) Mouse anti-GDF11, Santa Cruz Cat#sc-81952, 1:200; RRID: AB_2279007
3) Rabbit anti-GDF11, Abcam Cat#ab124721, 1:200; RRID: AB_10974143
4) Rabbit anti-GDF11, Novus Cat# NBP1-95888, 1:200; RRID: AB_11059784
5) Mouse anti-β-actin, Sigma-Aldrich Cat#A5441, 1:10000; RRID: AB_476744
6) Mouse anti-Myc, Cell Signaling Cat#2276, 1:2000; RRID: AB_331783
7) Guinea pig anti-NeuN Millipore Cat#ABN90, 1:1000; RRID: AB_11205592
8) Rabbit anti- CaMKIIα Abcam Cat#ab131468, 1:500; RRID: AB_11157799
9) Rabbit anti-GABA Sigma-Aldrich Cat#A2052, 1:500; RRID: AB_ 477652
10) Rabbit anti-Olig2 Millipore Cat#AB9610, 1:200; RRID: AB_570666
11) Chicken anti-GFAP Millipore Cat#AB5541, 1:1000; RRID: AB_177521
12) Rabbit anti-Iba1 WAKO Cat# 019-19741, 1:500; RRID: AB_ 839504
13) Goat anti-Doublecortin (DCX) Santa Cruz Cat#sc-8066, 1:1000; RRID: AB_ 2088494
14) Rabbit anti-p21 Waf1/Cip1 Cell Signaling Cat# 2947, 1:200; RRID: AB_ 823586
15) Rabbit anti Tmem159 Biorbyt Cat#orb351361, 1:1000;
16) Rabbit anti pSmad2 Cell Signaling Cat#3108, 1:1000; RRID: AB_490941
17) Rabbit anti Smad2 Cell Signaling, Cat#5339, 1:2000; RRID: AB_ 10626777
18) Rabbit anti Smad3 Cell Signaling, Cat#9513, 1:2000; RRID: AB_2286450
19) CyTM3 affinipure donkey anti-rabbit IgG (H+L) Jackson ImmunoResearch Cat#711-165-152, 1:500; RRID: AB_2307443
20) CyTM3 affinipure donkey anti- guinea pig IgG (H+L) Jackson ImmunoResearch Cat#706-165-148, 1:500; RRID: AB_2340460
21) CyTM3 affinipure donkey anti- chicken IgG (H+L) Jackson ImmunoResearch Cat#703-165-155, 1:500; RRID: AB_2340363
22) CyTM3 affinipure donkey anti-goat IgG (H+L) Jackson ImmunoResearch Cat#705-165-147, 1:500; RRID: AB_2307351
23) Alexa Fluor® 488 affinipure donkey anti-mouse IgG(H+L) Jackson ImmunoResearch Cat#715-545-151, 1:500; RRID: AB_2341099
24) Alexa Fluor® 488 affinipure donkey anti- guinea pig IgG(H+L) Jackson ImmunoResearch Cat#706-545-148, 1:500; RRID: AB_2340472
25) Alexa Fluor® 488 affinipure donkey anti-rabbit IgG (H+L) Jackson ImmunoResearch Cat#711-545-152, 1:500; RRID: AB_2313584
26) Peroxidase affinipure donkey anti-rabbit IgG (H+L) Jackson ImmunoResearch Cat#711-035-152, 1:500; RRID: AB_10015282
27) Peroxidase affinipure donkey anti-mouse IgG (H+L) Jackson ImmunoResearch Cat#715-035-151, 1:500; RRID: AB_2340771
28) Nanogold®-IgG goat anti mouse IgG (H+L) Nanoprobes, Cat# 2001-1.0 mL, 1:100; RRID: AB_2877644

Validation

All the primary antibodies in this paper were obtained from the manufacturer with validation and citations.
Validation of the antibodies used in this study:
1) Mouse anti-GDF11, R&D Systems Cat#MAB19581; RRID: AB_2877166. This antibody was systematically characterized in this study: see this paper in details. REF: PMID: 31133057. https://www.rndsystems.com/cn/products/human-gdf-11-bmp-11-antibody-743833_mab19581
2) Mouse anti-GDF11, Santa Cruz Cat#sc-81952; RRID: AB_2279007, REF: PMID: 30726519. https://www.scbt.com/p/gdf-11-antibody-x-19
3) Rabbit anti-GDF11, Abcam Cat#ab124721; RRID: AB_10974143, REF: PMID: 31148205. https://www.abcam.cn/products/primary-antibodies/gdf11--gdf8myostatin-antibody-epr45672-ab124721.html
4) Rabbit anti-GDF11, Novus Cat# NBP1-95888; RRID: AB_11059784. Application: immunofluorescence and immunohistochemistry in this paper. https://www.novusbio.com/products/gdf-11-bmp-11-antibody-epr4567-2-_nbp1-95888
5) Mouse anti-β-actin, Sigma-Aldrich Cat#A5441; RRID: AB_476744, REF: PMID17185421. https://www.sigmaaldrich.cn/CN/zh/product/sigma/a5441
6) Mouse anti-Myc, Cell Signaling Cat#2276; RRID: AB_331783, REF: PMID: 33372599. https://www.cellsignal.cn/products/primary-antibodies/myc-tag-9b11-mouse-mab/2276
7) Guinea pig anti-NeuN, Millipore Cat#ABN90; RRID: AB_11205592, REF: PMID32876565. https://www.sigmaaldrich.cn/CN/zh/product/mm/abn90

8) Rabbit anti- CaMKIIα, Abcam Cat#ab131468; RRID: AB_11157799, REF: PMID: 30387713. https://www.abcam.cn/products/primary-antibodies/camkii-alpha-antibody-ab131468.html
9) Rabbit anti-GABA, Sigma-Aldrich Cat#A2052; RRID: AB_ 477652, REF: PMID28445462. https://www.sigmaaldrich.cn/CN/zh/product/sigma/a2052
10) Rabbit anti-Olig2, Millipore Cat#AB9610; RRID: AB_570666, REF: PMID: 21782903. https://www.sigmaaldrich.cn/CN/zh/product/mm/ab9610
11) Chicken anti-GFAP, Millipore Cat#AB5541; RRID: AB_177521, REF: PMID: 27023006. https://www.sigmaaldrich.cn/CN/zh/product/mm/ab5541
12) Rabbit anti-Iba1, WAKO Cat# 019-19741; RRID: AB_ 839504, REF: PMID: 18022954. http://www.biodee.net/Show/index/cid/87/id/60470.html
13) Goat anti-Doublecortin (DCX), Santa Cruz Cat#sc-8066; RRID: AB_ 2088494, REF: PMID: 16786555. https://www.scbt.com/p/doublecortin-antibody-c-18?requestFrom=search
14) Rabbit anti-p21 Waf1/Cip1, Cell Signaling Cat# 2947;RRID: AB_ 823586, REF: PMID: 33360689. https://www.cellsignal.cn/products/primary-antibodies/p21-waf1-cip1-12d1-rabbit-mab/2947
15) Rabbit anti Tmem159, Biorbyt Cat#orb351361. https://www.biorbyt.com/tmem159-antibody-orb351361.html
16) Rabbit anti pSmad2, Cell Signaling Cat#3108; RRID: AB_490941, REF: PMID: 33149148. https://www.cellsignal.cn/products/primary-antibodies/phospho-smad2-ser465-467-138d4-rabbit-mab/3108
17) Rabbit anti Smad2, Cell Signaling, Cat#5339; RRID: AB_ 10626777, REF: PMID: 32928102. https://www.cellsignal.cn/products/primary-antibodies/smad2-d43b4-xp-rabbit-mab/5339
18) Rabbit anti Smad3, Cell Signaling, Cat#9513; RRID: AB_2286450, REF: PMID: 35766589. https://www.cellsignal.cn/products/primary-antibodies/smad3-antibody/9513

Eukaryotic cell lines

Policy information about cell lines and Sex and Gender in Research

Cell line source(s)	HEK293T from ATCC<br>Neuro-2a from ATCC
Authentication	All cell lines were validated based on the morphology and STR profiling before use.
Mycoplasma contamination	HEK293T and Neuro-2a cell lines are tested negative for mycoplasma contamination.
Commonly misidentified lines<br>(See ICLAC register)	No commonly misidentified cell lines were used in the study

Animals and other research organisms

Policy information about studies involving animals; ARRIVE guidelines recommended for reporting animal research, and Sex and Gender in Research

Laboratory animals	1) Aged of 3 months-old male mouse: C57BL/6J from Shanghai Slac Laboratory<br>2) Aged of 3 months, 9 months and 36 months-old male mouse: CD-1®(ICR) IGS from Zhejiang Academy of Medical Sciences<br>3) Mouse: GDF11f/f was originally generated in this paper<br>4) Mouse: CaMKIIα-Cre; GDF11f/f (GDF11cKO) obtained through crossing the two lines in this paper<br>5) Mouse: p21f/f was generated in this paper<br>6) Mouse: CaMKIIα-Cre; GDF11f/f ;p21f/f was generated in this paper<br>7) Two female cmmon marmosets (Callithrix jacchus, 260g aged 62M and 245g aged 70M) from Li-Xia Gao Lab in Zhejiang University.<br><br>Mice were housed in an environment of suitable temperature (25 °C) and humidity (typically 50%) under a 12 h light-dark cycle (light on from 7 a.m. to 7 p.m.) with accessing to food and water ad libitum.
Wild animals	The study did not involve wild animals.
Reporting on sex	Sex was not considered in this study design and methods.
Field-collected samples	This study did not involve samples collected from the field.
Ethics oversight	The studies with mice were approved by Medical Ethics Review Committee of Zhejiang University School of Medicine (ETHICS number: 14660).<br><br>The studies with cmmon marmosets were approved by Medical Ethics Review Committee of Zhejiang University School of Medicine (ETHICS number: ZJU20210053).

Note that full information on the approval of the study protocol must also be provided in the manuscript.
